# Structural Cement‐Based Supercapacitors with Multifunctional Robustness for Energy Storage

**DOI:** 10.1002/advs.202515769

**Published:** 2025-10-14

**Authors:** Qingyang Liu, Fengjuan Wang, Yu Zhang, Shuo Dong, Zhiyong Liu, Liguo Wang, Taotao Feng, Shiyu Sui, Yuncheng Wang, Jinyang Jiang, Peng Li

**Affiliations:** ^1^ School of Civil Engineering and Architecture Shandong University of Science and Technology Qingdao 266590 China; ^2^ School of Materials Science and Engineering Southeast University Nanjing 211189‌ China

**Keywords:** carbon cement electrode, CB‐hydrogel network, energy storage, structural supercapacitor

## Abstract

The rapid deployment of renewable energy demands cost‐effective and scalable energy storage solutions. While cement‐based supercapacitors offer transformative potential, their development is hindered by charge storage capacity, mechanical strength, and environmental stability. Herein, a breakthrough carbon cement supercapacitor (CCS) with exceptional electrochemical performance and excellent robustness is engineered. The porous carbon cement (CC) electrode, characterized by high strength, extremely low resistance, and high‐connectivity conductive hydrogel electrolyte, is prepared by thermomechanical consolidation at 90 °C. Through in situ polymerization around sodium dodecyl sulfate (SDS)‐mediated carbon black (CB) surfaces, a CB‐hydrogel network is built inside the multiscale pore structure of the carbon‐cement electrode. The CCS exhibits a leading areal capacitance (1708 mF cm^−^^2^), over 83% capacitance retention after 10 000 cycles, high strength (>8 MPa), 92.2% capacitance retention under extreme loading conditions, a wide operating temperature range from −20 to 80 °C with less than 9% capacitance fluctuation, and incombustibility. This new device exhibits potential to revolutionize energy‐storage systems.

## Graphical abstract

The breakthrough carbon cement supercapacitor features exceptional electrochemical performance and excellent robustness. The porous carbon cement electrode, characterized by high strength, extremely low resistance, is prepared by thermomechanical consolidation at 90 °C. Through in situ polymerization around sodium dodecyl sulfate‐mediated carbon black (CB) surfaces, a CB‐hydrogel network is built inside the multiscale pore structure of the cement electrode.

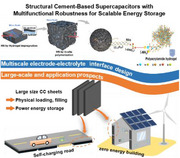

## 1 Introduction

Renewable energy supplies such as wind, solar, and tidal wave sources have typical time‐dependent characteristics, requiring mass‐scalable devices for energy storage to address the temporal mismatch between energy supplies and consumption. Massive energy storage using electrochemical batteries faces the challenge of mineral precursor scarcity.^[^ ^1^, ^2^, ^3^, ^4^ ^]^ Carbon cement supercapacitors (CCS) have the advantages of fast charge‐discharge efficiency and are capable of handling instantaneous high‐power demands.^[^ ^5^ ^]^ The electrodes of CCS can be easily prepared by a water and cement mixture doped with a relatively low concentration of carbon black (CB). Owing to the readily available and widely distributed nature of the raw materials, this approach offers a scalable and practical solution for energy storage. Notably, CCS can be made into structural components for large‐scale infrastructure, avoiding the occupation of limited space.^[^ ^6^, ^7^, ^8^ ^]^ Also, the integration of structure and energy storage provides innovative solutions for the construction of lunar infrastructure.^[^ ^9^ ^]^

At present, cement‐based energy devices, including CCS, commonly suffer from low energy density.^[^ ^10^, ^11^ ^]^ The energy storage mechanism of CCS relies on ionic adsorption via the electric double layer effect.^[^ ^12^, ^13^, ^14^, ^15^ ^]^ Increasing the CB amount can increase the number of energy storage sites and improve the capacitance,^[^ ^16^ ^]^ while significantly decreasing electrode strength. Physical compaction can reduce electrode resistance and increase strength, whereas it reduces porosity and capacitance.^[^ ^17^, ^18^ ^]^ Besides, an increase in electrode area can significantly improve both the capacitance and coulombic efficiency, while an electrode thickness increment can only increase the capacitance.^[^ ^19^ ^]^

Beyond CCS, emerging cement‐based energy devices employ cementitious materials as separators. While utilizing conventional commercial electrodes, these systems require ultra‐thin (< 1 cm), highly porous cement separators to facilitate efficient ion transport. Cells with cement separators exhibit substantially compromised electrochemical performance compared to commercial separators.^[^ ^20^ ^]^ Foamed concrete ^[^ ^21^ ^]^ and ice‐templating methods ^[^ ^22^, ^23^ ^]^ enable high‐porosity separator fabrication. However, it is still a challenge that increasing porosity would incur significant mechanical compromises.

Current cement‐based energy devices universally employ aqueous electrolytes, which suffer from electrolysis above 1.23 V, evaporative loss, and freezing susceptibility, disrupting ionic conductivity.^[^ ^24^, ^25^ ^]^ However, the large‐scale application of cement‐based energy devices limits their refined packaging. The evaporation is also enhanced by cement‐based materials’ inherent porosity.^[^ ^26^, ^27^ ^]^ The freezing of electrolytes at negative temperatures will render them unusable in many regions of the Earth. Conductive hydrogels provide a way to improve the stability of electrolytes.^[^ ^28^ ^]^

In this work, we proposed a structural CCS with multifunctional robustness for scalable energy storage via the design of carbon cement electrodes (CC) and hydrogel electrolyte, and optimized the interface interaction between them. The conductive hydrogel network across the CCS multiscale pore structure from nano to micro is generated through in situ polymerization and vacuum impregnation. An active agent is further introduced to promote the in situ polymerization of hydrogel monomer on the surface of CB. It makes full use of CB charge storage sites and enables the integration of multiscale hydrogel network (MSHN) and CB particles, significantly improving the energy density, cycle performance, and robustness of CCS. The designed CCS exhibits excellent robustness and can work normally under the conditions of bearing fracture, flame burning, and a wide temperature range of −20–80 °C.

## 2 Results and Discussion

### 2.1 Design Strategy and Preparation

**Figure 1:**
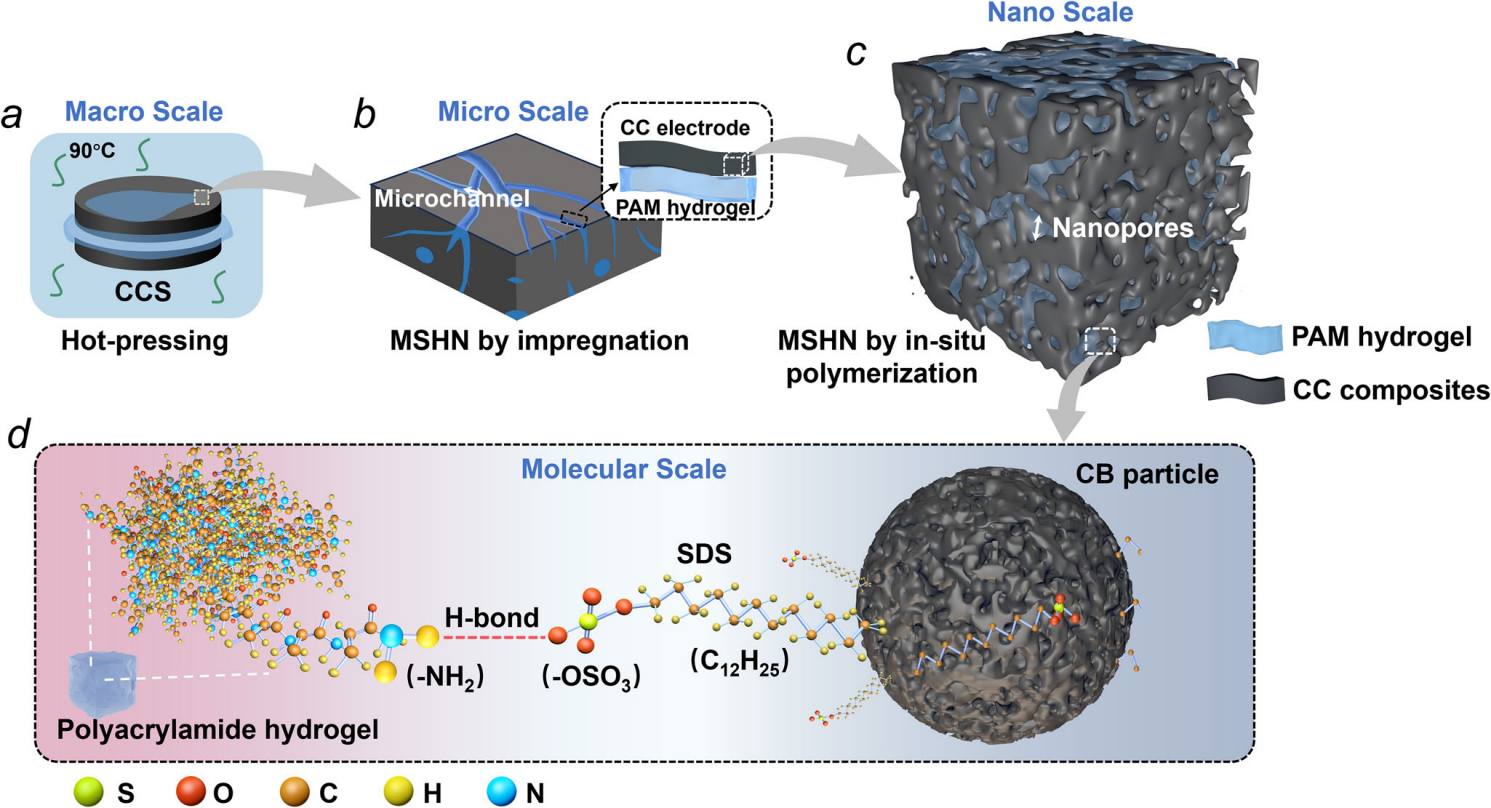
Multi‐scale design of CC electrode, involving a) hot‐pressing preparation and building a MSHN from microscale to nanoscale via b) hydrogel impregnation and c) in situ polymerization. d) At the molecular scale, SDS acts as the bridge promoting the in situ polymerization of PAM monomers around the CB surface.

This study enhances the mechanical strength, electrochemical performance, and robustness of CCS through the following three synergistic strategies, as illustrated in **Figure** **1**, achieving concurrent optimization of these key performances. The sample name is defined in Table 1. Detailed experimental methods and procedures are given in Section S1 (Supporting Information).

**Figure 2:**
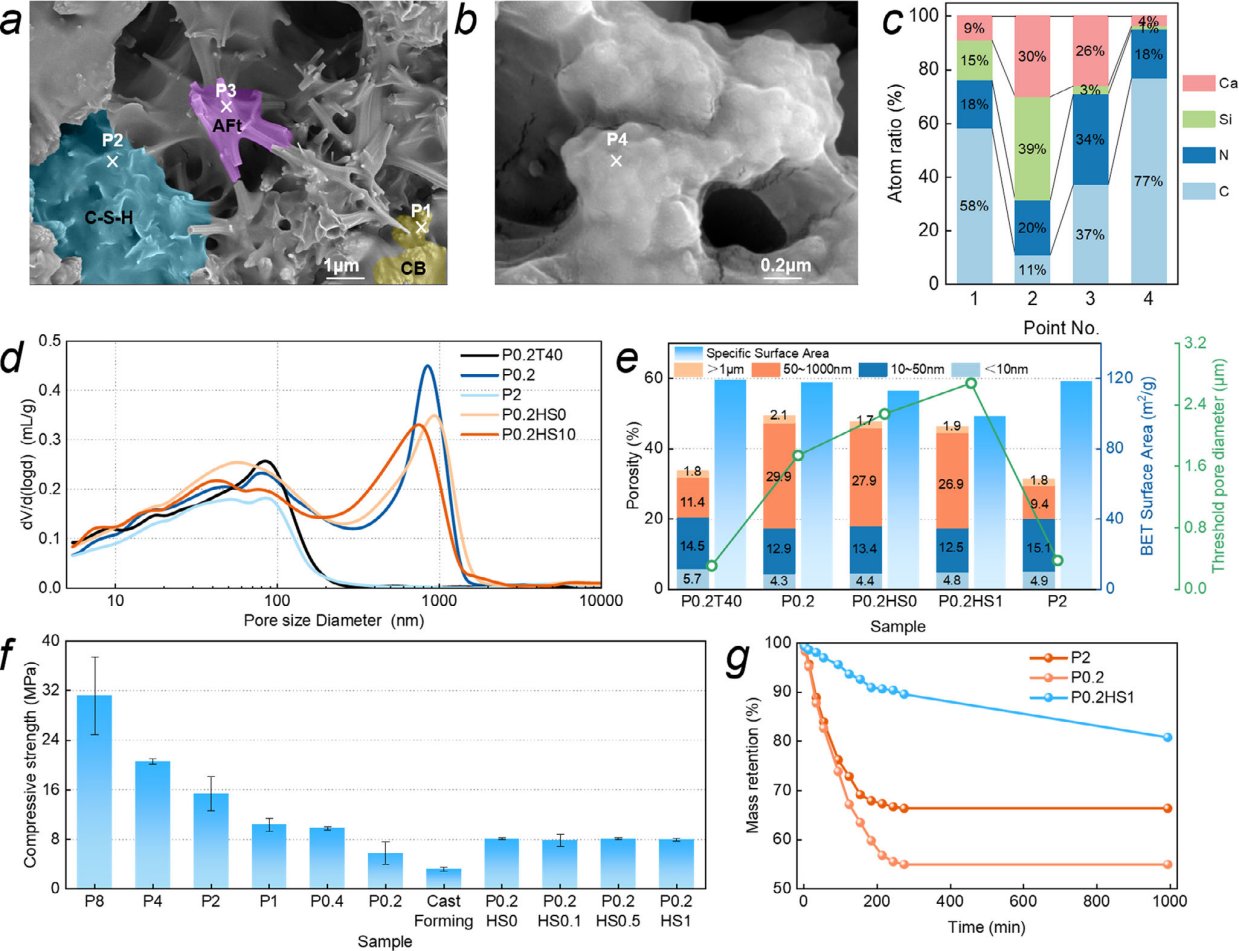
Microstructure and physical features of CC electrode: a,b) morphology, c) chemical compositions, d) pore size distribution, e) porosity and specific surface area, f) compressive strength, and g) mass evolution subject to 60 °C oven at the unpacked state.

**Figure 3:**
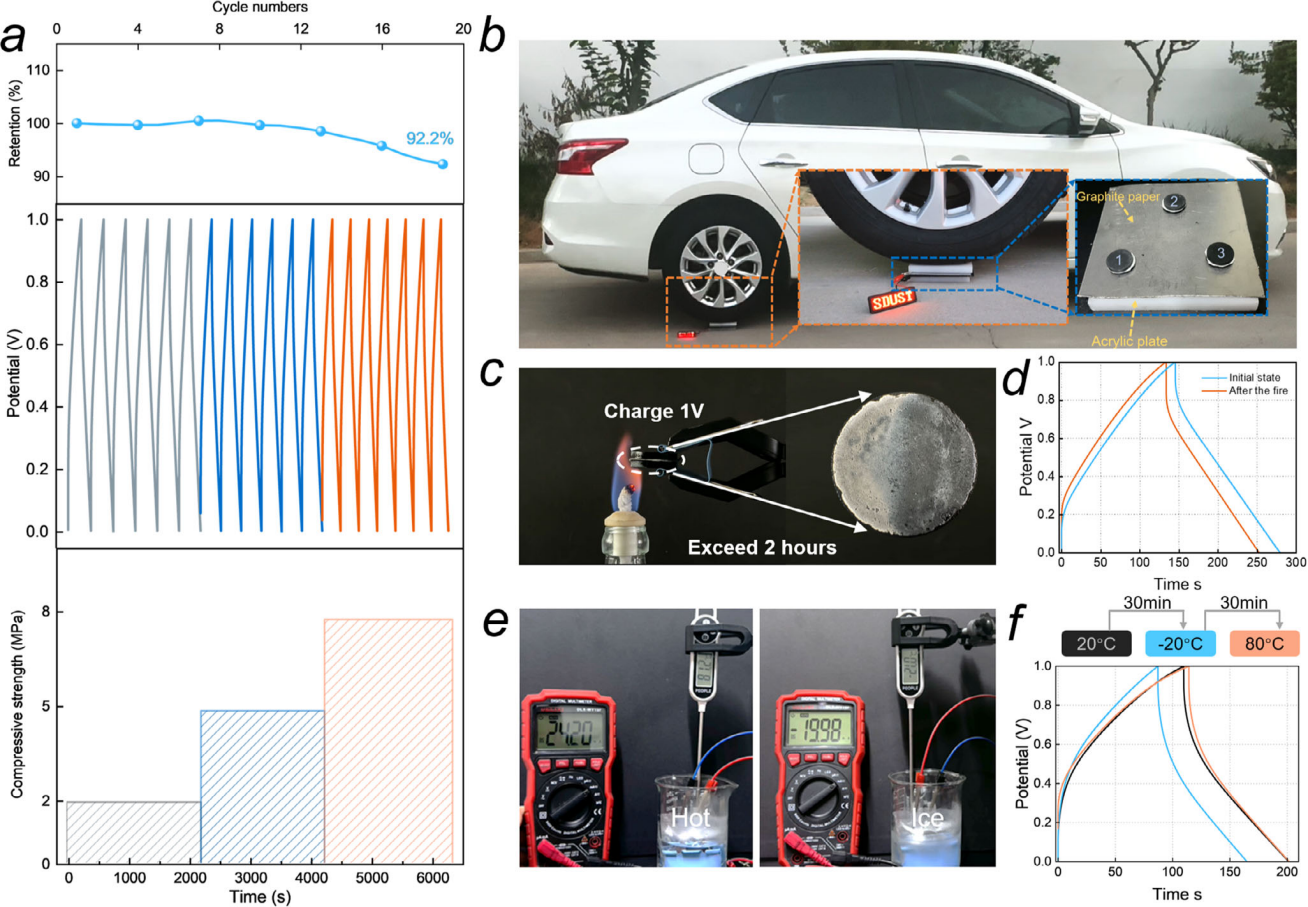
Multi‐scenario application of CCS: a) GCD curve of the CCS at the loaded condition, b) photograph of three parallel connected 4.9 cm^2^ devices illuminating a light‐emitting diode under compressive load. c,d) combustion test and the GCD curve in 10 mA cm^−2^ after combustion test, e,f) application at −20 and 80 °C, and the GCD curve in 10 mA cm^−2^.

**Figure 4:**
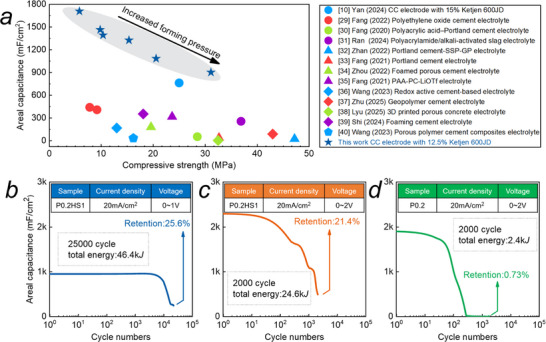
Capacitance, strength, and cycling performance: a) comparison of CCS with the reported results published in the past five years, in terms of capacitance and compressive strength,^[^ ^16^, ^29^, ^30^, ^31^, ^32^, ^33^, ^34^, ^35^, ^36^, ^37^, ^38^, ^39^, ^40^ ^]^ and b–d) cycling performances at different cycling numbers and voltage windows. Details involving the electrolyte and electrode used in the references are listed in Table S6 (Supporting Information).

**Figure 5:**
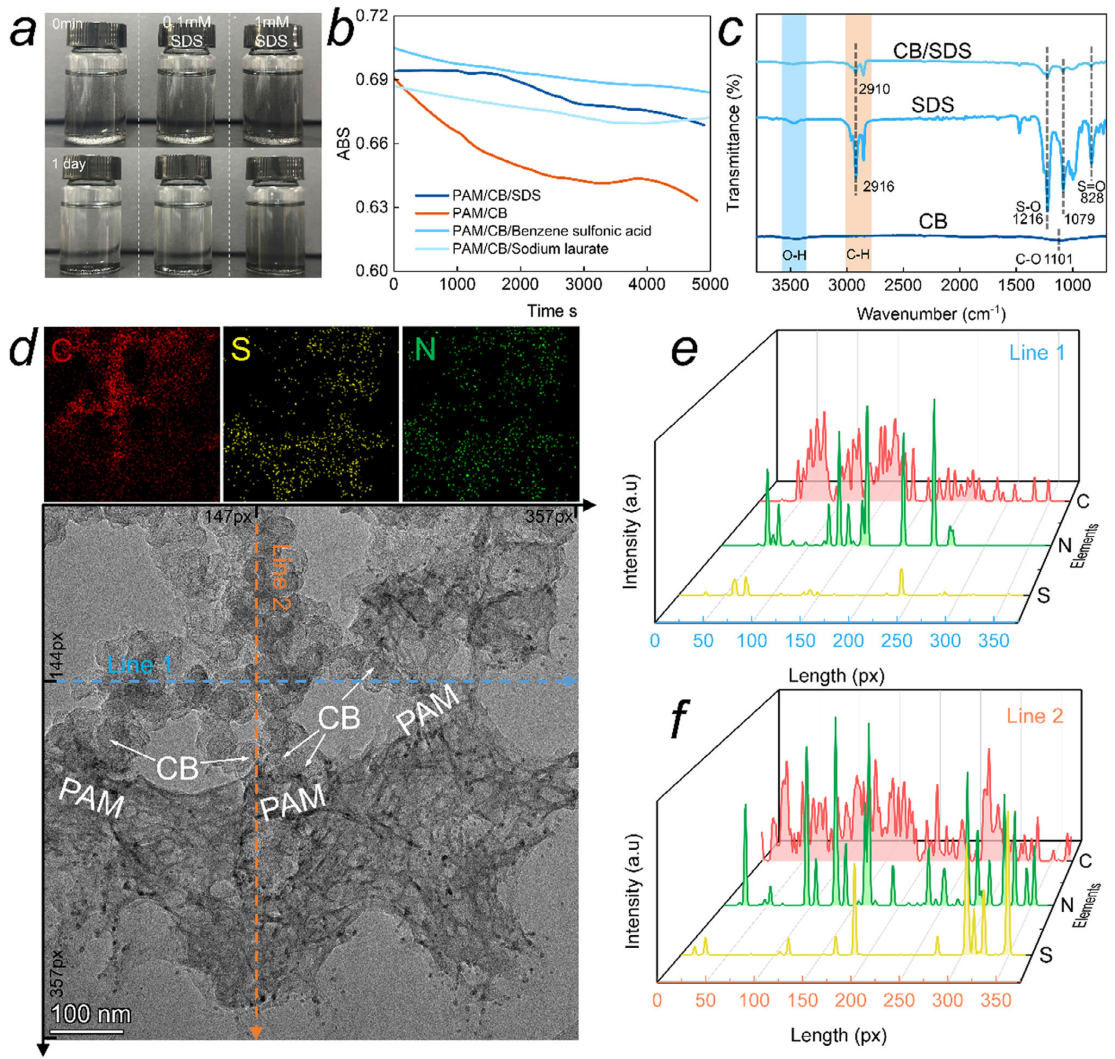
SDS effect on the affinity between CB with PAM hydrogel: a) effect of different content SDS on the CB dispersion in the acrylamide monomer solution, b) absorbance values of CB ultrasonic suspension solution, c) FTIR results, d) TEM image and energy dispersive spectrometer‌ (EDS) mapping, e,f) EDS line scanning of line 1 and 2 in the TEM image.

**Figure 6:**
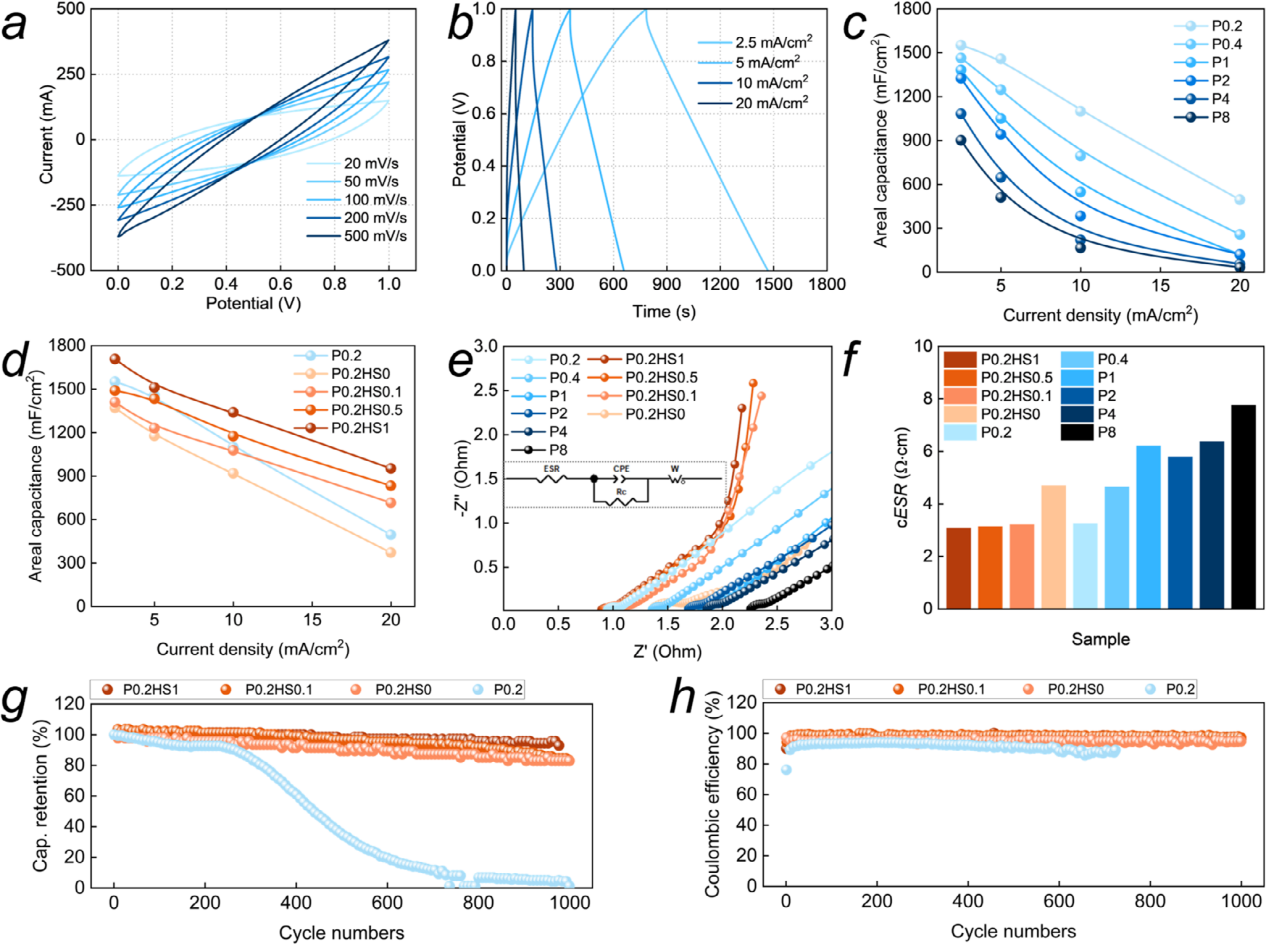
Electrochemical behavior of CCS: a,b) CV and GCD curves of P0.2HS1, c,d) areal capacitance as functions of forming pressure and SDS addition, e,f) Nyquist plots and equivalent series resistance (*ESR*), g,h) capacitance retention and coulombic efficiency during cycling.

1) Thermomechanical consolidation for structural‐electrical synergy. High‐temperature physical compaction (90 °C, 0.2–8t) reduces the volume by 30.4% (Figure S13, Supporting Information) and the mean interparticle spacing of CB aggregates by 27.8% (Figure S14, Supporting Information). Simultaneously, it increases electrode strength by 9.68 times, from 3.2 to 31 MPa (**Figure** **2**f), and lowers the dried CC electrodes’ resistance by 64% (Figure S15, Supporting Information). 90 °C environment promotes moisture motions, which avoids pore channels closure under high compaction (Figure 2d). This endows the porous electrode with numerous interconnected sub‐micro pores (Figure 2d) and high transport efficiency of the internal electrolyte (Figure S16, Supporting Information).

2) Multiscale hydrogel electrolyte network. Hierarchical electrolyte networks from molecular to microlevel are built within the electrode porous structure via the mixture with acrylamide monomer during preparation and the vacuum impregnation of polyacrylamide (PAM) hydrogel after hardening. It achieves 90% water retention after 200 min drying at 60 °C oven at the state of unpackage (Figure 2g), suppresses electrolyte freezing down to −20 °C (**Figure** **3**f), maintains >83% capacitance after 10 000 cycles (**Figure** **4**b), and makes high‐voltage window working possible (Figure 4c,d).

3) Interface‐optimized CB‐hydrogel integration. Hydrophobic CB particles are modified by an amphiphilic active agent, such as sodium dodecyl sulfate (SDS), in this work. Meanwhile, in situ polymerization of PAM monomers around the CB surface is conducted, so that the SDS would take as bridges to connect the CB and PAM. It significantly promotes the affinity between PAM hydrogel and CB (**Figure** **5**a) and avoids electrolyte‐depleted zones at the interfaces. It builds a CB‐hydrogel network structural integration in the electrode (Figure 5d). The resistivity of the electrode full of KCl electrolyte drops from 4.8 to 3 Ω·cm (**Figure** **6**f). The capacitance of CCS is enhanced by 156%, from 371 to 952 mF cm^−2^ in 20 mA cm^−2^ of current density (Figure 6d).

**Table 1:** Sample name abbreviation.

Sample	Forming pressure	Temperature	Hydrogel electrolyte	Surfactant	Surfactant content
Casting formation	0	90 °C	–	–	–
P0.2T40	0.2t	40 °C	–	–	–
P0.2	0.2t	90 °C	–	–	–
P0.4	0.4t	90 °C	–	–	–
P1	1t	90 °C	–	–	–
P2	2t	90 °C	–	–	–
P4	4t	90 °C	–	–	–
P8	8t	90 °C	–	–	–
P0.2HS0	0.2t	90 °C	Yes	–	–
P0.2HS0.1	0.2t	90 °C	Yes	SDS	0.1mm
P0.2HS0.5	0.2t	90 °C	Yes	SDS	0.5mm
P0.2HS1	0.2t	90 °C	Yes	SDS	1mm

### 2.2 Interfacial Compatibility Between CB and Hydrogel Molecules

It is the poor interfacial affinity between hydrophobic CB and PAM hydrogel, resulting in the rapid agglomeration and precipitation of CB (Figure 5a). Surfactant molecules, such as SDS, benzene sulfonic, and sodium laurate, contribute to the excellent dispersion of CB in the PAM hydrogel. Absorbance of CB/acrylamide solution with surfactant decreases much slower than the reference (Figure 5b). SDS exhibits a better effect and ensures that CB particles remain suspended for more than one day. The surfactant containing both hydrophobic and hydrophilic molecular sides takes as a bridge and facilitates good interfacial compatibility between CB and hydrogel molecules.

FTIR analysis of vibrational characteristics confirms the interaction between the surfactant and CB particles. CB contains only a small amount of hydrophilic functional groups of C─O (1101 cm^−1^) and O─H (3435 cm^−1^),^[^ ^41^ ^]^ which accounts for the hydrophobic behavior. SDS is rich in hydrophilic groups of S─O (1079, 1216 cm^−1^) and S═O (828 cm^−1^), as well as hydrophobic long‐chain alkyl groups.^[^ ^42^, ^43^ ^]^ After forming the SDS‐CB composite, the C─H stretching vibration peak of SDS at 2916 cm^−1^ undergoes a leftward shift of 6 cm^−1^, accompanied by a noticeable change in peak shape. This shift is attributed to the interaction between the long alkyl chains of SDS and the surface of the CB, which affects the vibrational characteristics of the C─H bond.^[^ ^44^, ^45^ ^]^ Other functional group peaks show no significant change.

The TEM images illustrate the affinity between modified CB and PAM hydrogel (Figure 5d). CB particles are well wrapped by the wrinkled PAM.^[^ ^46^, ^47^ ^]^ In Figure 5e,f, the element distribution profiles show the overlapping among S, N, and C elements, which correspond to SDS/PAM and CB, respectively. The highly overlapping of characteristic elements means the overlapping of matters.

### 2.3 Microstructure and Physical Performances of CC Electrodes

CC electrode microstructure plays roles in both two effects, namely, taking as the storage space of electrolyte and the carrier for bonding CB particles. Microstructure and physical features of the CC electrode are shown in Figure 2 in terms of morphology, microstructure, strength, and water holding capacity.

The microscale morphology of the CC electrode is a multi‐phase composite system, including CB particles and the hydrates of C‐S‐H gel and ettringite (Figure 2a). Figure 2b shows the microstructural morphology of CB encapsulated within the hydrogel at higher magnification. The composites are wrapped by a layer of dried hydrogel film, featuring a large area and flat surface. The wrapping has no selectivity for any type of substance. The high connectivity and large‐area hydrogel networks would provide electrolyte storage space and transport channels.

CC electrode microstructure exhibits the multiscale and porous features, which endow it with a large specific surface area of more than 110 g cm^−2^ (Figure 2d,e). The CC electrode has a porosity of up to 50%, which is composed of pores with a size range from nano to micro. Pore size distribution profile of P0.2 sample, prepared by 90 °C and 0.2 t hot‐press method, shows a bimodal feature at the pore size of ≈50 and 810 nm. As the forming temperature decreases to 40 °C, however, the bimodal distribution transforms into a single one at the pore size of ≈80 nm. The higher temperature of 90 °C promotes moisture motion and evaporation, which avoids pore channels closure under high compaction. It creates numerous sub‐micro pores (Figure 2d), which would promote ion transport efficiency.

As forming pressure rises to 2 t or more, compared with the P0.2 sample, the pore size distribution profile exhibits a single peak at ≈80 nm. The threshold pore size sharply decreases to 150 nm. Submicro pores with a pore volume of 29.9% can be eliminated by mechanical compaction. Higher compaction squeezes out more moisture from electrodes, and also inhibits moisture motion and connective pore channel formation.

Specific surface area is little sensitive to the forming compaction pressure, maintaining at 118 m^2^ g^−1^. The specific surface area and porosity of the CC electrode can be diminished by the addition of PAM hydrogel and SDS. SDS addition can endow the CB particles with hydrophilic features (Figure 5a), and promote their uniform distribution and facilitate the growth and deposition of hydrates/hydrogels at the interface. It avoids electrolyte‐depleted zones at the interfaces of porous CB. As a result, SDS addition decreases the conductivity of the electrode full of KCl electrolyte from 4.8 to 3 Ω·cm (Figure 6f).

In terms of strength, it significantly depends on the forming pressure (Figure 2f). It is 31 MPa at the forming pressure of 8 t, and decreases to 3.2 MPa with the forming pressure reduction to zero. The P0.2 sample fabricated via 90 °C hot‐pressing under 2 t pressure exhibits comparable porosity to cast‐formed samples (Figure S17, Supporting Information), while achieving a 2.5‐fold enhancement in compressive strength (from 3.2 to 8.0 MPa). This method enables synergistic optimization of mechanical robustness and porous microstructure.^[^ ^48^ ^]^

The formation of MSHN in the P0.2HS0 sample has little impact on the microstructure of CC electrodes, while leading to an increase by 33% in compressive strength. This is attributed to the nano reinforcement of highly‐connected PAM polymer.^[^ ^49^, ^50^, ^51^ ^]^

Water holding capacity of the CC electrode underlies the long‐term application of the CC supercapacitor. It can be characterized by the mass evolution of unpacked CC electrodes at 60 °C (Figure 2g). For the samples P1 and P0.2, water in the electrode quickly evaporates, and it is totally dried after 270 min exposure, with mass retention of 54.9% and 66.3%. In contrast, the mass retention of P0.2HS1 is still 80.7% after 1000 min. The hydrogel network in the CC electrode significantly improves the capacity of water holding.

### 2.4 Electrochemical Performance of CCS

Electrochemical performances and capacitance measurements of the CCS are conducted upon conventional electrochemical cyclic voltammetry (CV) and galvanostatic charge‐discharge (GCD) cycles (Figure 6a,b). The CV curve at a scanning rate of 20–500 mV s^−1^ is shuttle‐shaped with well symmetry. It indicates that the mechanism of its charge storage is physical adsorption based on the electric double layer. The full shuttle shape represents good storage performance. In the GCD test, a selected current of 2.5–20 mA cm^−2^ range is applied and maintained constant until reaching a target voltage of 1 V, at which point the current density is instantaneously reversed until 0 V potential difference. The GCD curve approximates a symmetrical triangle, exhibiting well charge/discharge reversibility. Both have high sensitivity to discharge/charge energy density. As current density increases, the calculated capacitance decreases significantly (Figure 6c,d).

The Nyquist plots of different groups at open‐circuit voltage (OCV) by using electrochemical impedance spectroscopy tests, as depicted in Figure 6e. The profiles resemble a semicircle at the very beginning and then develop along diagonal line. The equivalent series resistance (*ESR*) of CCS, reflecting the transfer loss caused by internal resistance, can be obtained from Nyquist plots. The *ESR* can reach 3 Ω·cm (P0.2HS1) (Figure 6f), and it enables a symmetrical triangle of the GCD curve, which means extremely low energy consumption due to the low internal resistance.

*ESR* rises from 3.2 to 7.8 Ω·cm with an increase in the forming pressure from 0.2 to 8 t (Figure 6f). This counterintuitive trend arises from porosity reduction, which disrupts ionic percolation networks and limits electrolyte transport, despite a 27.8% decrease in the average interparticle distance of CB aggregates that lowers the dry electrode's electrical resistance (Figure S15, Supporting Information). Correspondingly, rising forming pressure decreases the capacitance of CCS (Figure 6c). Taking the current density case of 2.5 mA cm^−2^ as an example, the capacitance at 0.2 t can be up to 1551 mF cm^−2^, and it gradually reduces by 41.6%, to 901 mF cm^−2^ at 8 t. Forming pressure rising increases the compactness and strength of the CC electrode, while decreases pore volume and increases tortuosity. It reduces electrolyte amount and extends the ion transport path.

With MSHN formation, *ESR* increases from 3.3 Ω·cm (P0.2) to 4.7 Ω·cm (P0.2HS0) (Figure 6f). This result is consistent with the results of dry electrode resistance (1.15 Ω·m → 2.53 Ω·m) (Figure S15, Supporting Information). Inherent hydrophobicity of CB impedes hydrogel electrolyte wetting, creating localized interfacial delamination and electrolyte‐depleted zones at CB/hydrogel interfaces. It disrupts continuous ion transport networks essential for electrochemical performance. This problem can be optimized by SDS. The *ESR* of P0.2HS1 decreases to 3 from 4.7 Ω·cm of P0.2HS0 (Figure 6f).

The capacitance of CCS exhibits the same trend as *ESR*, that is, it decreases with MSHN generation and improves by SDS addition (Figure 6d). Taking the current density of 20 mA cm^−2^ as an example, the capacitance of P0.2 is 494 mF cm^−2^. It decreases to 371 mF cm^−2^ as MSHN generation (P0.2HS0). SDS addition increases the capacitance by 156% and it reaches to 952 mF cm^−2^ (P0.2HS1).

Capacitance retention and coulombic efficiency during cycling work is shown in Figure 6g,h. As compared with P0.2 using aqueous electrolyte, P0.2HS0 with MSHN electrolyte shows significantly improved cycling stability at a current density of 10 mA cm^−^^2^, with stable charging and discharging for over 100 h. After 1000 cycles, the capacitance retention rate reaches 82.9%. The capacitance retention rate of P0.2HS1 further increases to 92.9%, and the coulombic efficiency remains above 99%. The P0.2 exhibits rapid capacitance decay after 240 cycles, primarily due to water evaporation from the electrolyte and insufficient contact between the electrode and electrolyte, which leads to a rapid loss of double‐layer capacitance.

Performance comparisons with related references are shown in Figure 4. The references include almost all reports on cement‐based supercapacitors or cement electrochemical cells in the past 5 years, include electrode parameter, areal capacitance, compressive strength, energy density and cycles performance, to the best of our knowledge. The details are presented in Table S6 (Supporting Information). Areal capacitance and strength are the vital parameters for the structural energy‐storage devices, while they are generally contradictory with each other. Electrodes’ porous structure containing electrolyte or conductive substances underlies good electrochemical performance, but decreases the strength. The maximum capacitance in our work can be up to 1708 mF cm^−2^ with a compressive strength of 8 MPa, which is large enough to the load bearing of subgrade and infilled walls.

CCS cycling performance can be up to 5000 cycles with the capacitance retention higher than 95%, and the capacitance retention at 10 000 cycles is also higher than 83% (Figure 4b). This is attributed to the excellent physical stability of the electrolyte in the multiscale hydrogel network. Furthermore, the capacitance retention at 25 000 cycles decreases to 25% (Figure 4b). Soaking the electrode in the electrolyte again can slightly restore its performance from 25% to 45%. Then it can still work in the subsequent 10 000 cycles, and the capacitance retention decreases to 23% (Figure S26, Supporting Information). Salts deposition on the CB surface may be a critical factor. It shows the ultra‐long cycling performance.

Notably, MSHN can also significantly improve the cycling performances at high voltage of 2 V. The cycling number at which the capacitance retention declines to 1000 mF cm^−2^ increases significantly from 100 to 1700. Also, the stored energy during all life cycle improves by more than ten times, from 2.4 J (P0.2) to 24.6 J (P0.2HS1).

### 2.5 Excellent Robustness for Structural Supercapacitors

CCS exhibits robustness in the environment of loading, fire, and extreme temperature, as depicted in Figure 3. The electrochemical performances of CCS are insensitive to loading lower than the ultimate strength. As the load rises to near the ultimate strength (8 MPa), the electrode periphery peels off and cracks, whereas the capacitance retention decreases slightly to 92.2% (Figure 3a). The CCS can still work normally under three through cracks and light up a bulb (Figure S27, Supporting Information). Figure 3b gives a photograph of three parallel connected 4.9 cm^2^ devices illuminating a light‐emitting diode under compressive load.

The alcohol lamp continuously burns the electrode for 2 h, as shown in Figure 3c. During firing, the electrode volume is stable, and there has been no combustion or explosion. Part of the electrolyte is burned and evaporated. After soaking and replenishing the electrolyte again, the electrochemical performance of CCS can be almost restored (Figure 3d).

Electrochemical performance at a wide range of temperatures from −20 to 80 °C is depicted in Figure 3e,f. A simple package is shown in Figure S28 (Supporting Information). CCS is charged to 1 V, and then placed in a freezer. When the thermometer displays −20 °C, GCD testing is performed. Then the CCS is placed in an environment of 80 °C for GCD testing. Low temperature limits the ion motions in the electrolyte, leading to a slight capacitance decay of less than 9%. High temperature has little impact on capacitance.

### 2.6 Large‐Scale Scalability of CC Structural Supercapacitors

Benefiting from the cement‐based matrix, cement‐based electrodes exhibit simple preparation and widely available raw materials. The hot‐pressing method is also applicable to the fabrication of large‐volume specimens, indicating the potential for industrial‐scale production. The influence of size expansion on electrochemical performance has been previously investigated, showing that increasing electrode thickness leads to reduced specific capacitance and lower energy utilization efficiency, whereas enlarging the electrode area can effectively enhance the overall capacity. With their exceptional robustness and tunable mechanical strength, cement‐based electrodes hold great promise for future applications in smart low‐carbon infrastructures such as zero‐energy buildings and self‐charging roads (Figure S30, Supporting Information).

## 3 Conclusion

We engineered a novel cement‐based structural supercapacitor from these aspects: electrode microstructure, electrolyte, and electrode‐electrolyte interface. The supercapacitor exhibits leading capacitance (1708 mF cm^−2^), >10 000 charge‐discharge cycles, well strength (>8 MPa), 92.2% capacitance retention under the conditions of critical load, has wide working temperature range (−20–80 °C), and excellent thermal safety under direct flame exposure.

High‐temperature compaction (90 °C, 0.2–8 t) increases electrode strength by 9.68 times, from 3.2 to 31 MPa, and reduces the mean interparticle spacing of CB particles by 27.8%, and so lowers CB network resistance. Notably, 90 °C environment promotes moisture motions, which creates numerous sub‐micro pores. This method enables synergistic optimization of mechanical robustness and porous microstructure.

MSHN not only promotes the physical stability of electrolytes, but also increases anti‐electrolysis ability. It includes the performances of water retention, capacitance fluctuation <9% at −20 °C, maintaining 83% capacitance after 10 000 cycles, and an improvement in the stored energy of all life cycle at 2 V by more than 10 times, from 2.4 to 24.6 J.

In situ polymerization based SDS bridging promotes the growth of hydrogel molecules on the CB and eliminates electrolyte‐depleted zones around the hydrophobic CB interfaces. It is shown by the further drops in the resistance of the electrode full of KCl electrolyte and a significant increment in capacitance.

## Conflict of Interest

The authors declare no conflict of interest.

## Supporting information



Supporting Information

## Data Availability

The data that support the findings of this study are available from the corresponding author upon reasonable request.

## References

[1] J. Rissman , C. Bataille , E. Masanet , N. Aden , W. R. Morrow , N. Zhou , N. Elliott , R. Dell , N. Heeren , B. Huckestein , J. Cresko , S. A. Miller , J. Roy , P. Fennell , B. Cremmins , T. Koch Blank , D. Hone , E. D. Williams , S. de la Rue du Can , B. Sisson , M. Williams , J. Katzenberger , D. Burtraw , G. Sethi , H. e. Ping , D. Danielson , H. Lu , T. Lorber , J. Dinkel , J. Helseth , Appl. Energy 2020, 266, 114848.

[2] X. Zhang , X. Cheng , Q. Zhang , J. Energy Chem. 2016, 25, 967.

[3] J. Mitali , S. Dhinakaran , A. A. Mohamad , Energy Storage and Saving 2022, 1, 166.

[4] C. Zhao , W. Dong , T. M. Indra Mahlia , L. Shi , K. Wang , S. P. Shah , W. Li , Energy Build. 2025, 338, 115732.

[5] N. Chanut , D. Stefaniuk , J. C. Weaver , Y. Zhu , Y. Shao‐Horn , A. Masic , F. J. Ulm , Proc. Natl. Acad. Sci. USA 2023, 120, 2304318120. 10.1073/pnas.2304318120 PMC10410735 37523534

[6] H. Zhou , H. Li , L. Li , T. Liu , G. Chen , Y. Zhu , L. Zhou , H. Huang , Mater. Today Energy 2022, 24, 100924.

[7] L. Zeng , P. Li , Y. Yao , B. Niu , S. Niu , B. Xu , Materials Today Nano 2020, 12, 100094.

[8] C. Zhao , W. Dong , J. Liu , S. Peng , W. Li , Cem. Concr. Compos. 2025, 163, 106165.

[9] M. Z. Naser , Acta Astronaut. 2019, 155, 264.

[10] N. Shirshova , H. Qian , M. S. P. Shaffer , J. H. G. Steinke , E. S. Greenhalgh , P. T. Curtis , A. Kucernak , A. Bismarck , Composites, Part A 2013, 46, 96.

[11] D. Shan , J. Yang , W. Liu , J. Yan , Z. Fan , J. Mater. Chem. A 2016, 4, 13589.

[12] P. Sharma , T. S. Bhatti , Energy Convers. Manage. 2010, 51, 2901.

[13] H. Ji , X. Zhao , Z. Qiao , J. Jung , Y. Zhu , Y. Lu , L. i. Zhang , A. H. MacDonald , R. S. Ruoff , Nat. Commun. 2014, 5, 3317. 24557361 10.1038/ncomms4317

[14] N. S. Choi , Z. Chen , S. A. Freunberger , X. Ji , Y. K. Sun , K. Amine , G. Yushin , L. F. Nazar , J. Cho , P. G. Bruce , Angew. Chem., Int. Ed. 2012, 51, 9994. 10.1002/anie.201201429 22965900

[15] M. R. Lukatskaya , B. Dunn , Y. Gogotsi , Nat. Commun. 2016, 7, 12647. 27600869 10.1038/ncomms12647 PMC5023960

[16] D. Yan , J. Mao , R. Gao , W. Wang , S. Wang , S. Ruan , H. Qian , F. Mu , S. Chen , Y. Liu , J. Energy Storage 2024, 96, 112717.

[17] Z. Wu , H. Pan , P. Huang , J. Tang , W. She , Adv. Mater. 2024, 36, 2405183. 10.1002/adma.202405183 38973222

[18] L. Wei , W. Zuo , H. Pan , K. Lyu , W. Zhang , W. She , Composites, Part B 2021, 226, 109333.

[19] P. Li , Q. Liu , J. Jiang , F. Wang , Y. Zhang , J. Energy Storage 2025, 130, 117446.

[20] Z. Liu , P. Feng , R. Liu , L. Yuan , X. Meng , G. Tao , J. Chen , Q. Ran , J. Hong , J. Liu , C. Miao , Natl. Sci. Rev. 2024, 11, nwae309. 39355271 10.1093/nsr/nwae309 PMC11444079

[21] A. Raj , D. Sathyan , K. M. Mini , Constr. Build. Mater. 2019, 221, 787.

[22] Y. Wang , Y. Zheng , W. Li , S. Xiao , S. Chen , J. Xing , C. Xiong , Y. Zhou , W. Zhang , T. Hihara , N. Moloto , C. Miao , Sci. Bull. 2025, 70, 1994. 10.1016/j.scib.2025.03.032 40180853

[23] Q. Cai , J. Guo , R. Zhang , Z. Li , P. Feng , J. Luo , X. Zhao , L. Xu , K. Wu , Adv. Mater. 2025, 37, 2505202. 10.1002/adma.202505202 40534302

[24] W. G. Nunes , B. G. A. Freitas , R. M. Beraldo , R. M. Filho , L. M. Da Silva , H. Zanin , Sci. Rep. 2020, 10, 19195. 33154430 10.1038/s41598-020-75851-7 PMC7644765

[25] C. Zhao , W. Dong , K. Wang , Z. Tao , W. Li , Constr. Build. Mater. 2025, 481, 141612.

[26] X. Zhang , M. Du , H. Fang , M. Shi , C. Zhang , F. Wang , Constr. Build. Mater. 2021, 299, 124290.

[27] Y. u. Zhang , J. Wu , L. Wang , G. Sun , D. Hou , J. Yu , J. Jiang , J. Am. Ceram. Soc. 2024, 107, 5022.

[28] C. Lu , H. Jiang , X. Cheng , J. He , Y. Long , Y. Chang , X. Gong , K. Zhang , J. Li , Z. Zhu , J. Wu , J. Wang , Y. Zheng , X. Shi , L. Ye , M. Liao , X. Sun , B. Wang , P. Chen , Y. Wang , H. Peng , Nature 2024, 629, 86. 38658763 10.1038/s41586-024-07343-x

[29] C. Mani‐Lata , C. Hussakan , G. Panomsuwan , J. Composites Science 2020, 4, 121.

[30] J. D. Hines , J. Colloid Interface Sci. 1996, 180, 488.

[31] J. Zhang , Y. Qi , T. Lv , X. Niu , B. Tai , J. Mater. Res. Technol. 2023, 24, 1706.

[32] P. Zhang , G. Qian , Z. P. Xu , H. Shi , X. Ruan , J. Yang , R. L. Frost , J. Colloid Interface Sci. 2012, 367, 264. 22137168 10.1016/j.jcis.2011.10.036

[33] C. M. González‐García , M. L. González‐Martín , R. Denoyel , A. M. Gallardo‐Moreno , L. Labajos‐Broncano , J. M. Bruque , Carbon 2005, 43, 567. 10.1016/j.jcis.2004.06.012 15450441

[34] L. Shen , H. Ding , Q. Cao , W. Jia , W. Wang , Q. Guo , Carbon 2012, 50, 4284.

[35] Z. Hu , G. Chen , Adv. Mater. 2014, 26, 5950. 24923256 10.1002/adma.201400179

[36] Y. u. Zhang , Q. Liu , L. Wang , D. Hou , J. Jiang , ACS Appl. Mater. Interfaces 2025, 17, 32752. 40392104 10.1021/acsami.4c22154

[37] D. Hou , J. Xu , Y. u. Zhang , G. Sun , Composites, Part B 2019, 177, 107421.

[38] K. Wu , J. Long , L. Qing , G. De Schutter , Constr. Build. Mater. 2024, 432, 136542.

[39] Y. u. Zhang , L. e. Guo , J. Shi , Q. Luo , J. Jiang , D. Hou , Cem. Concr. Res. 2022, 161, 106964.

[40] C. Fang , D. Zhang , Electrochim. Acta 2022, 401, 139491.

[41] C. Fang , D. Zhang , J. Mater. Chem. A 2020, 8, 12586.

[42] M. Ran , J. Wang , D. Zhang , J. Energy Storage 2024, 79, 110169.

[43] P. Zhan , J. Xu , J. Wang , J. Zuo , Z. He , Cem. Concr. Compos. 2023, 137, 104924.

[44] C. Fang , D. Zhang , Constr. Build. Mater. 2021, 285, 122897.

[45] C. Zhou , Q. Wang , C. Zhang , Materials 2022, 15, 2459.35407792

[46] C. Fang , D. Zhang , Chem. Eng. J. 2021, 426, 130793.

[47] J. Wang , P. Zhan , D. Zhang , Cem. Concr. Compos. 2023, 138, 104987.

[48] J. i.‐H. Zhu , X. Wang , H. Yu , S. Liu , C. Pei , F. Xing , Cem. Concr. Compos. 2025, 161, 106106.

[49] Q. Lyu , Y. Wang , D. Chen , S. Liu , J. Mbabazi , P. Zhu , J. Lu , S. Wang , F. Yin , Cem. Concr. Compos. 2025, 157, 105926.

[50] M. Shi , D. Zhang , J. Power Sources 2024, 616, 235135.

[51] J. Wang , C. Xu , D. Zhang , P. Zhan , J. Energy Storage 2023, 63, 106991.

