# Supplementary material for: Structural Cement‐Based Supercapacitors with Multifunctional Robustness for Energy Storage
Source: Adv Sci (Weinh). 2025 Oct 14;13(2):e15769. doi: 10.1002/advs.202515769 (PMC12786344; doi:10.1002/advs.202515769)
Supplement: Supplementary file 1 — Supporting Information [file ADVS-13-e15769-s001.docx]

**Structural Cement-Based Supercapacitors with Multifunctional Robustness for Energy Storage**

*Qingyang Liu^1^*, *Fengjuan Wang^2^, Yu Zhang^1^,*, Shuo Dong^1^, Zhiyong Liu^2^, Liguo Wang^2^, Taotao Feng^2^, Shiyu Sui^2^, Yuncheng Wang^2^, Jinyang Jiang ^2^*, Peng Li^1^**

*1 School of Civil Engineering and Architecture, Shandong University of Science and Technology, Qingdao, China*

*2 School of Materials Science and Engineering, Southeast University, Nanjing, China*

**Corresponding author, E-mail:* [*tgyuzhang@outlook.com*](mailto:tgyuzhang@outlook.com)*;* [*jiangjinyang16@163.com*](mailto:jiangjinyang16@163.com)*;* [*lipeng@sdust.edu.cn*](mailto:lipeng@sdust.edu.cn)*.*

**S1. Preparation of CC electrode**

**Materials**

Conductive carbon black (CB, Ketjen600JD) was purchased from LION Corporation, Japan, and exhibited a specific surface area of 1342.41 m²/g based on BET analysis. The chemical composition of ordinary Portland cement is listed in Table S1. Polycarboxylate superplasticizer was employed to improve the workability of the cement slurry. Acrylamide was used as the monomer. Ammonium persulfate is the initiator. N,N′-methylenebisacrylamide is the cross-linker. And TMEDA is the catalyst. All analytical-grade reagents were obtained from Macklin Biochemical Co., Ltd. The conductivity of the conductive graphite paper was less than 0.005 S/cm. The glass fiber membrane had the thickness of 0.29 mm, the pore diameter of 1.63 µm, and the porosity greater than 90%.

**Electrode Preparation (Figure S1a, b):**

Polyacrylamide solution was prepared by mixing acrylamide monomer (5 wt% of cement mass) with the initiator, cross-linker, and catalyst, followed by the addition of water and then stirring. The ratio of the monomer, initiator, cross-linker, and catalyst was listed in Table S2. The initial solution exhibited a viscosity of 1.7 mPa·s, sufficient for basic mixing operations. CB powder was mixed with sodium dodecyl sulfate (SDS). The prepared polyacrylamide solution was then mixed with the CB/SDS composite to yield a CB slurry, which was subsequently mixed with ordinary Portland cement and stirred for 15 minutes to uniform mixing. The mix proportions are listed in Table S3. In addition to SDS, the surfactants of sodium laurate and benzenesulfonic acid were also investigated and the mix proportions was presented in Table S4.

Above prepared carbon cement (CC) slurry of 3.15 g was poured into a custom-designed tablet mold (25 mm in diameter). The specimens were pressed using a hydraulic tablet press. Upon reaching the target pressure, the load was maintained for 5 minutes before demolding. Then the specimens were tightly wrapped by plastic wraps and placed in the oven at 90°C for 12 hours. Subsequently, the specimens were cured under standard conditions (20 °C, 95% RH) for 28 days.

**Hydrogel Electrolyte (Figure S1c):**

The cured electrode sheets were vacuum-dried at 60 °C for 72 hours to remove the residual moisture in the pores. The dried electrode sheets were immersed in the polyacrylamide-KCl solution and subjected to vacuum impregnation at 0.1 atm for 48 hours. The concentration of KCl is 1 mol/L, and the mix proportion of polyacrylamide is listed in Table S2. Multiscale hydrogel electrolyte network was achieved through both in-situ polymerization and vacuum impregnation.


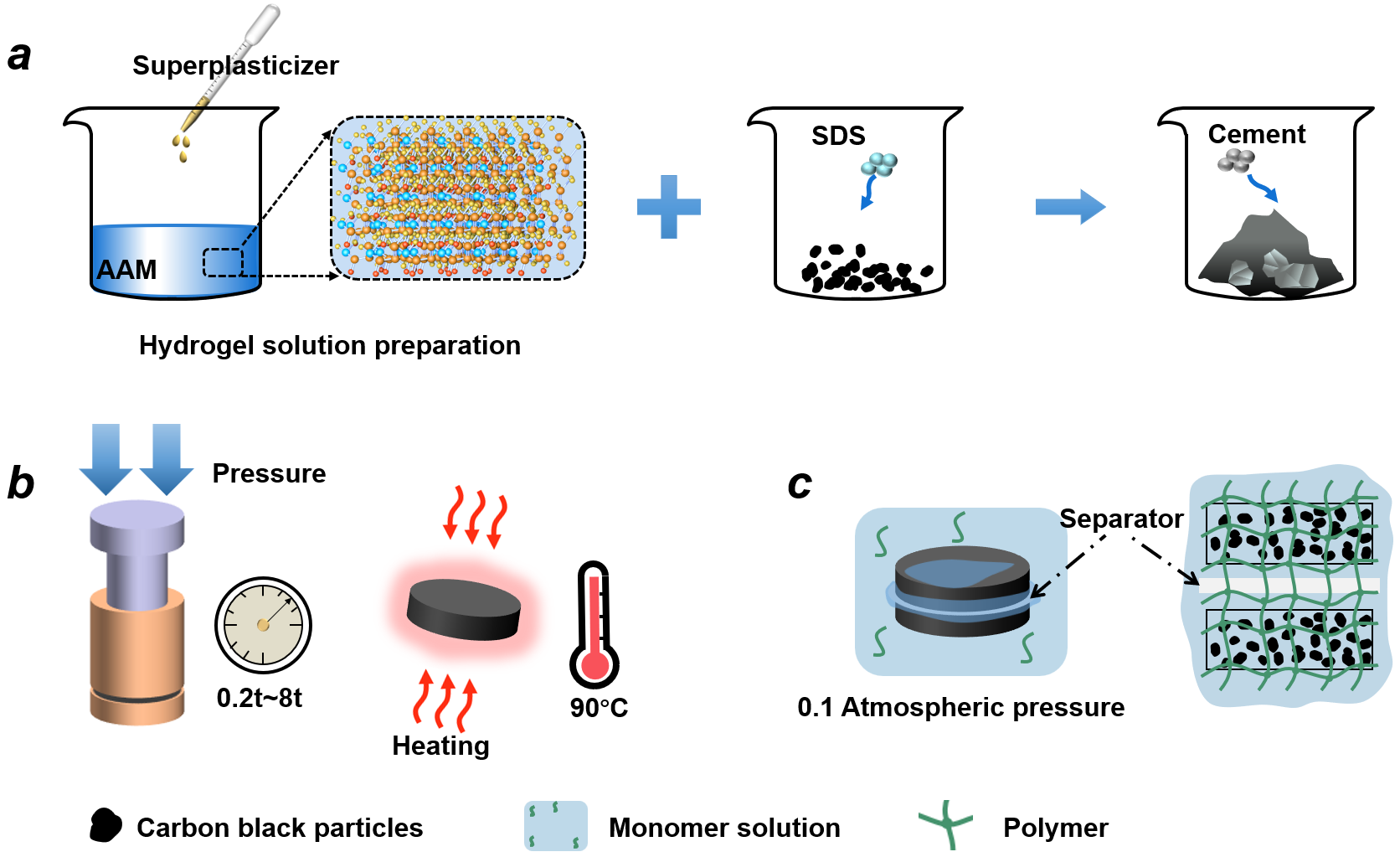


**Figure S1.** Preparation of carbon cement supercapacitors.

**Table S1 Chemical composition of cement (wt.%).**

| CaO | SiO_2_ | Al_2_O_3_ | SO_2_ | Fe_2_O_3_ | Surface area (m^2^/kg) |
| --- | --- | --- | --- | --- | --- |
| 64.3 | 21.5 | 6.7 | 5.6 | 4.5 | 368 |

**Table S2 Mix proportions of polyacrylamide solution**

|  | Acrylamide (%) | Ammonium persulfate (%) | Methylene diacrylamide (%) | TMEDA |
| --- | --- | --- | --- | --- |
| Polyacrylamide | 5 | 1.2 | 0.6 | 0.15 |

Note: The 5% is mass fraction of acrylamide monomer in cement, the other is the mass fraction of medicine in acrylamide monomer.

**Table S3 Mix proportions of CC electrode**

| Sample | Shaping pressure | *N*_CB_ (%) | W/C (%) | SP (g) | Hydrogel electrolyte | Surfactant | Surfactant content |
| --- | --- | --- | --- | --- | --- | --- | --- |
| Casting formation | 0 | 12.5 | 1.6 | 0.5 |  |  |  |
| P0.2T40 | 0.2t | 12.5 | 0.6 | 0.2 |  |  |  |
| P0.2 | 0.2t | 12.5 | 0.6 | 0.2 | - | - | - |
| P0.4 | 0.4t | 12.5 | 0.6 | 0.2 | - | - | - |
| P1 | 1t | 12.5 | 0.6 | 0.2 | - | - | - |
| P2 | 2t | 12.5 | 0.6 | 0.2 | - | - | - |
| P4 | 4t | 12.5 | 0.6 | 0.2 | - | - | - |
| P8 | 8t | 12.5 | 0.6 | 0.2 | - | - | - |
| P0.2HS0 | 0.2t | 12.5 | 0.6 | 0.2 | Yes | - | - |
| P0.2HS0.1 | 0.2t | 12.5 | 0.6 | 0.2 | Yes | SDS | 0.1mM |
| P0.2HS0.5 | 0.2t | 12.5 | 0.6 | 0.2 | Yes | SDS | 0.5mM |
| P0.2HS1 | 0.2t | 12.5 | 0.6 | 0.2 | Yes | SDS | 1mM |

Note: *N*_CB_ refers to the mass ratio of CB to cement, W/C refers to water/cement ratio, SP refers to the superplasticizer.

**Table S4 Mix proportions of the CC electrode with different surfactant**

|  | Cement | *N*_CB_ (%) | Water (%) | Superplasticizer (g) | Shaping pressure (t) | Surfactant |
| --- | --- | --- | --- | --- | --- | --- |
| P0.2HL0.1 | 1 | 12.5 | 0.6 | 0.2 | 0.2 | L-0.1mM |
| P0.2HL1 | 1 | 12.5 | 0.6 | 0.2 | 0.2 | L-1mM |
| P0.2HB0.1 | 1 | 12.5 | 0.6 | 0.2 | 0.2 | B-0.1mM |
| P0.2HB1 | 1 | 12.5 | 0.6 | 0.2 | 0.2 | B-1mM |

Note: L refers to sodium laurate, B refers to benzene sulfonic acid

**S2. Experiment characterization**

**UV-Vis Absorbance Test:**

To evaluate the dispersion stability of SDS-mediated CB in the polyacrylamide solution, Ultraviolet-Visible Spectroscopy (UV-Vis) spectrophotometer was employed to conduct dynamic absorbance measurements. The CB suspension was prepared by initially mixing Ketjen600JD CB with SDS, followed by dispersing the mixed powder into a polyacrylamide solution. The CB concentration was maintained at 0.5 wt.%. The resulting mixture was subjected to ultrasonic dispersion at 40 kHz for 30 minutes. Absorbance (Abs) was continuously monitored at a wavelength of 800 nm using a quartz cuvette for 80 minutes, with data collected at 3-second intervals.

**FTIR Analysis:**

The polyacrylamide-based CB suspension was vacuum-dried at 60 °C for 72 hours, then the dried powder was ground into the finer one that can pass through 200-mesh sieve. To investigate the surface modification effect of SDS on CB, the Ketjen600JD CB was mixed with SDS in dried powder form. Fourier Transform Infrared Spectroscopy (FTIR) transmittance spectra were collected using a Thermo Fisher Scientific Nicolet iS20 spectrometer.

**Raman spectroscopy Analysis:**

Carbon skeleton structure evolution of CB before and after SDS modification was estimated via Raman spectrometer by HORIBA LabRAM HR Evolution.

**XPS Analysis:**

X-ray photoelectron spectroscopy (XPS) analysis was conducted on CB powder, SDS powder, and dried PAM-CB suspension samples. The PAM-CB suspension was prepared by ultrasonically dispersing CB particles uniformly into a PAM solution. After gelation, the samples were dried in a vacuum oven at 60 °C for 72 hours. The dried samples were then ground into powder passing through a 200-mesh sieve for testing. XPS measurements were performed using a K-Alpha spectrometer (Thermo Scientific, USA).

**SEM Characterization:**

Scanning electron microscopy (SEM) was employed to characterize the morphology of the prepared electrode materials. A specimen with size of 5 mm × 5 mm × 5 mm were sectioned from the electrode and vacuum-dried at 60 °C for 72 hours to prevent surface carbonization. The fracture section of the sectioned specimen was observed by SEM using a ZEISS Sigma 360 SEM (Germany).

**TEM Characterization:**

The suspension was prepared by adding 0.3 wt% CB particles (containing 1 mM SDS) into the PAM solution, followed by ultrasonic treatment for 10 min to ensure uniform dispersion. A small amount of the resulting suspension was then dropped onto a copper grid and allowed to dry naturally at room temperature. Transmission electron microscopy (TEM) observations were conducted using an FEI Talos F200S microscope (Thermo Fisher Scientific, US).

**Porous Structure Analysis:**

To analyze the porous structure of electrode materials, porosity and pore size distribution were measured using an automated mercury intrusion porosimeter (Micromeritics AutoPore V 9620, USA). Additionally, the specific surface area of the electrode materials was determined by nitrogen adsorption porosimeter (Micromeritics ASAP 2460, USA).

**Resistivity Measurement:**

The resistance of the CC electrode sheets was measured using a conventional digital multimeter. Prior to testing, the electrode sheets were dried at 60 °C for 72 hours. Graphite papers were placed on both sides of the electrode sheets and secured with a specialized clamping device (Figure S2). The resistance across the graphite papers was measured, and the resistivity was calculated using the following formula:

$\rho=R\cdot\frac{\text{A}}{H}$ (1)

where $\rho$ is the resistivity (Ω·m), $R$ is the resistance measured by the multimeter (Ω), $H$ is the thickness of the electrode sheet (cm), and $\text{A}$ is the cross-sectional area (cm²).


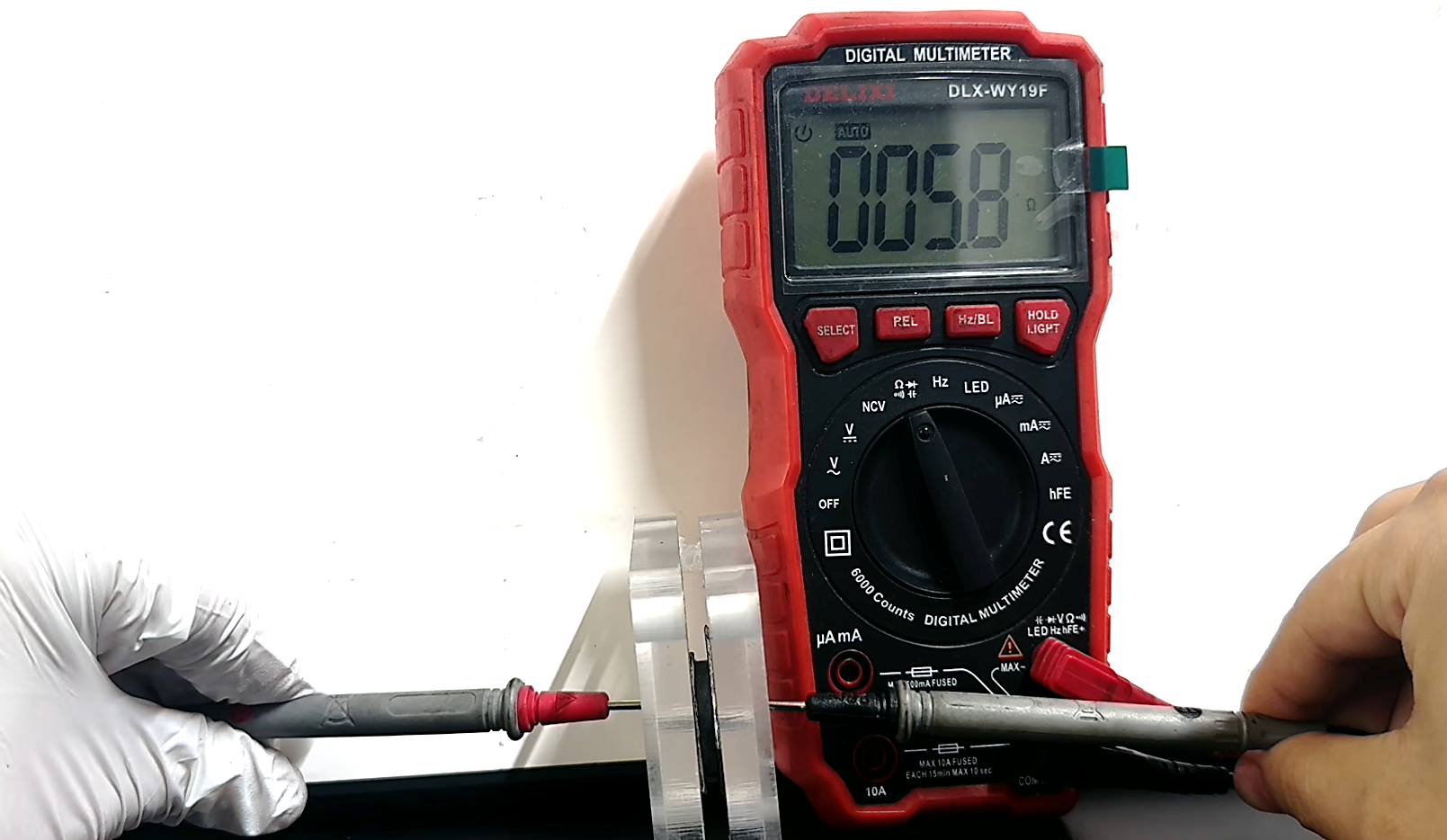


**Figure S2.** Method of resistivity test.

**Compressive Strength Test:**

Specimens with a diameter of 25 mm and a height of 20 mm were prepared for compressive strength testing. The tests were conducted using an MTS universal testing machine (model ETM304C) under displacement-controlled loading at a rate of 5 mm/min until specimen failure. The peak load $F$was recorded, and the compressive strength $f_{c}$ was calculated accordingly.

$f_{c} =\frac{F}{\text{A}}$ (2)

**Electrochemical Test:**

All electrochemical tests were conducted at the indoor environment. A CHI 760E electrochemical workstation (Chenhua, China) equipped with a two-electrode system was used to perform cyclic voltammetry (CV), galvanostatic charge-discharge (GCD), and electrochemical impedance spectroscopy (EIS).

CV measurements were carried out within a potential window of 0–1 V at five different scan rates: 20, 50, 100, 200, and 500 mV/s. GCD tests were conducted over the same voltage window (0–1 V) at the current density of 2.5, 5, 10, and 20 mA/cm².

EIS tests were conducted at open circuit potential across a frequency range of 0.01 Hz to 100 kHz. The impedance data were fitted using ZView software, with the fitting errors kept below 20%. The areal specific capacitance $C_{areal}$ was calculated according to the following equation:

(3)

where $I_{0}$ is the current density (A/cm²), $t_{dis}$ is the discharge time (s), $\text{A}$ is the electrode area (cm²), and $\text{U}_{0}$ is the potential window (V). The integral capacitance $C_{areal,CV}$ in the cyclic voltammetry (CV) test was calculated using the following equation:

(4)

where $u$ is the scan rate. The energy density $E$ (Wh/m³) and power density $P$ (W/m³) were calculated using the following equations:

$E=\frac{A\cdot C_{areal}\cdot\left(\Delta\text{U}\right)^{2}}{7200\cdot V}$ (5)

$P=\frac{E\cdot3600}{t_{dis}}$ (6)

where $\Delta\text{U}$ is the discharge voltage window, and $V$ is the volume of the capacitor.

**S3. Supplementary materials**

**S3.1 Raw material testing**


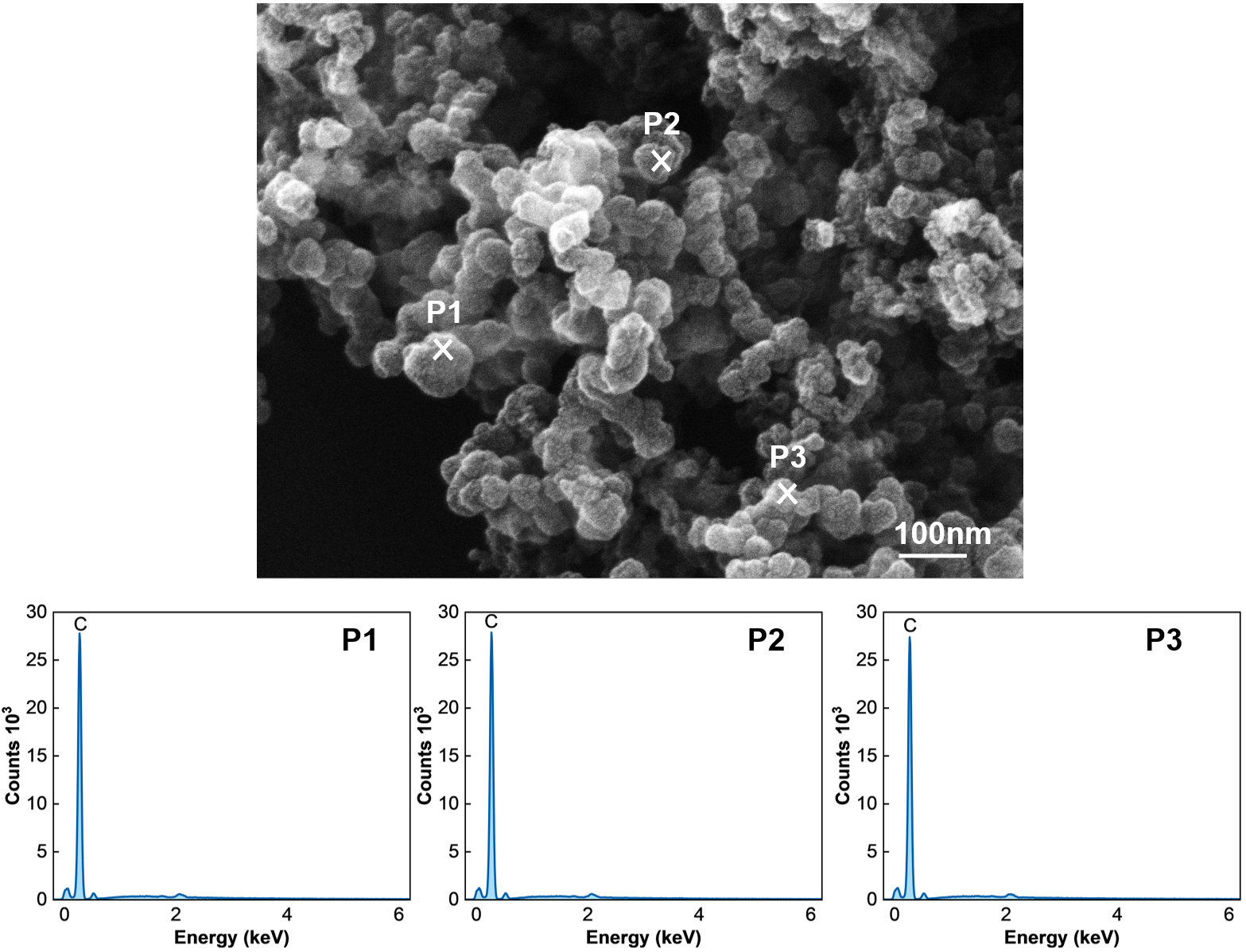


**Figure S3.** Micro morphology and chemical composition of CB. The morphology of the CB appears as irregular spheres with diameters of several tens of nanometers, exhibiting a pronounced clustering behavior. Point elemental analysis conducted on the surfaces of three randomly selected CB particles revealed the predominant carbon content, generally larger than 90%, with only trace amounts of other elements.


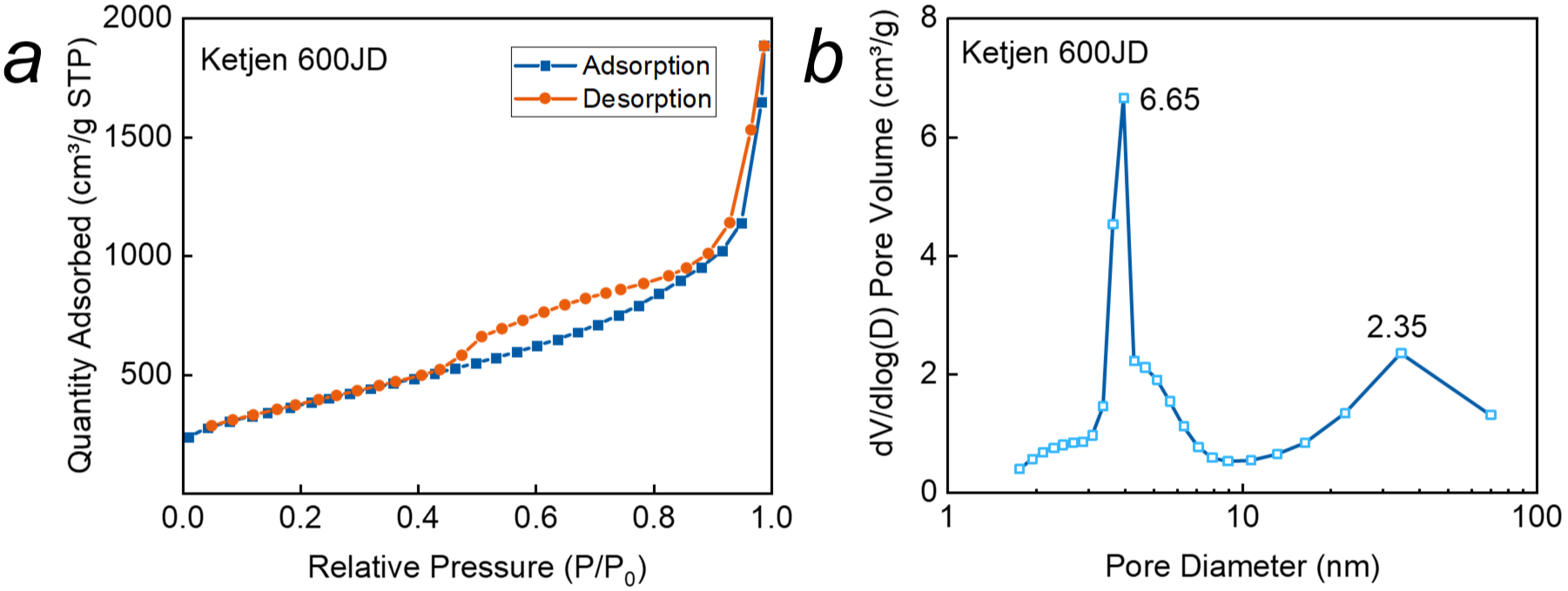


**Figure S4.** BET analysis results of CB. (a) Adsorption-desorption curves of CB. An obvious hysteresis loop is observed at relative pressures above 0.5, indicating the abundant porous features that contribute to a high specific surface area. (b) Pore size distribution of CB. The most probable pore diameter is approximately 4 nm [1, 2].


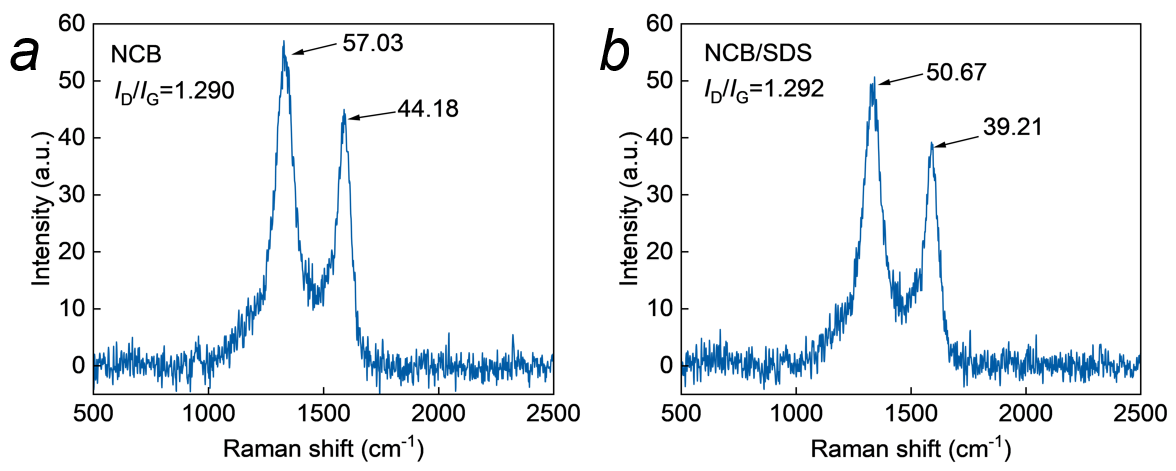


**Figure S5.** Raman spectra analysis results. (a) Raman spectrum of CB. The D and G bands located at approximately 1350 cm⁻¹ and 1580 cm⁻¹ correspond to structural defects and the degree of graphitization, respectively [3, 4]. The intensity ratio (*I*_D_/*I*_G_) of CB is 1.290, indicating a high degree of structural disorder and a predominantly amorphous carbon structure. (b) Raman spectrum of CB after SDS modification. It shows the minor shifts in peak positions and negligible changes in the *I*_D_/*I*_G_ ratio, suggesting that the SDS treatment does not significantly affect the lattice structure of CB.


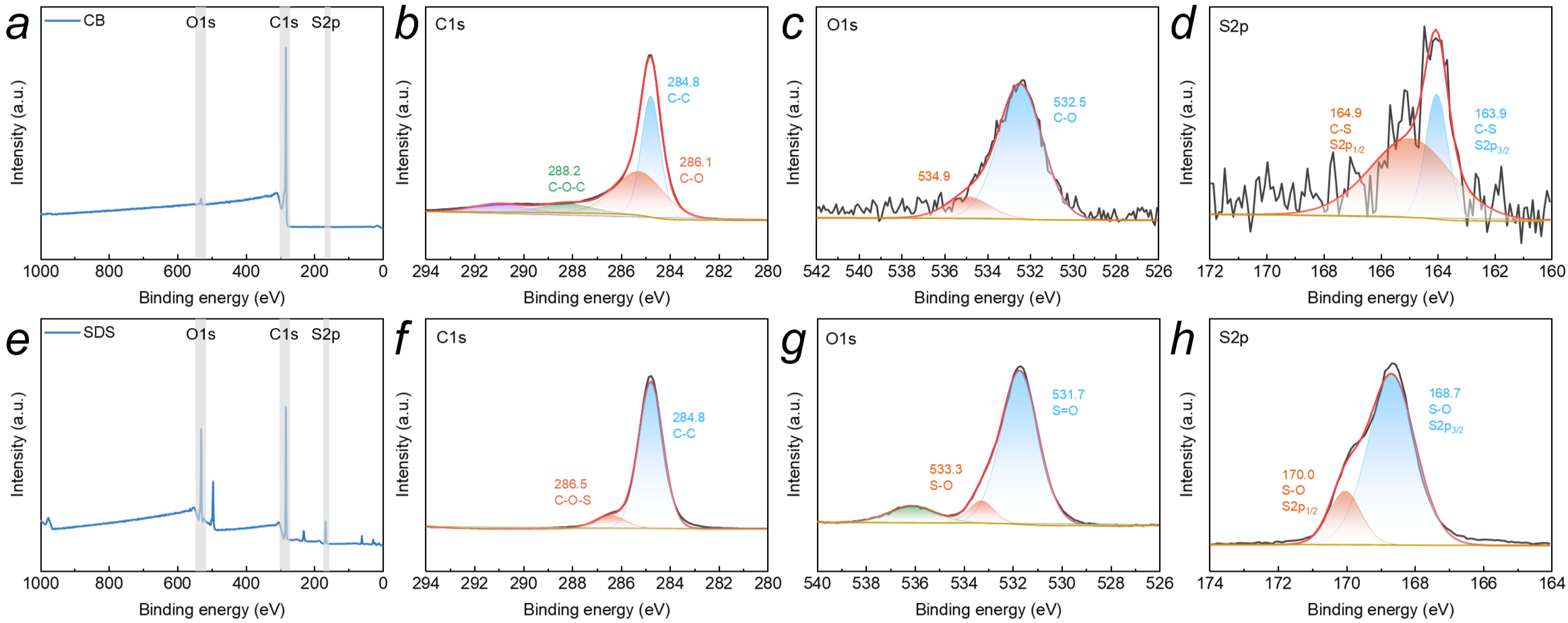


**Figure S6.** XPS spectra of CB and SDS. (a) Survey spectrum of CB. A strong C1s peak is near a binding energy of 284 eV, along with a weak O1s peak around 532 eV [5]. The S2p signal is below the detection limit. (b–d) High-resolution spectra of C1s, O1s, and S2p, respectively [6, 7]. (e) Survey spectrum of SDS, involving the distinct peaks of C1s, O1s, and S2p. (f–h) High-resolution spectra of C1s, O1s, and S2p in SDS, respectively. The S2p signal displays a characteristic doublet at binding energies of 170.0 and 168.7 eV.

**S3.2 CC electrode-electrolyte interface**


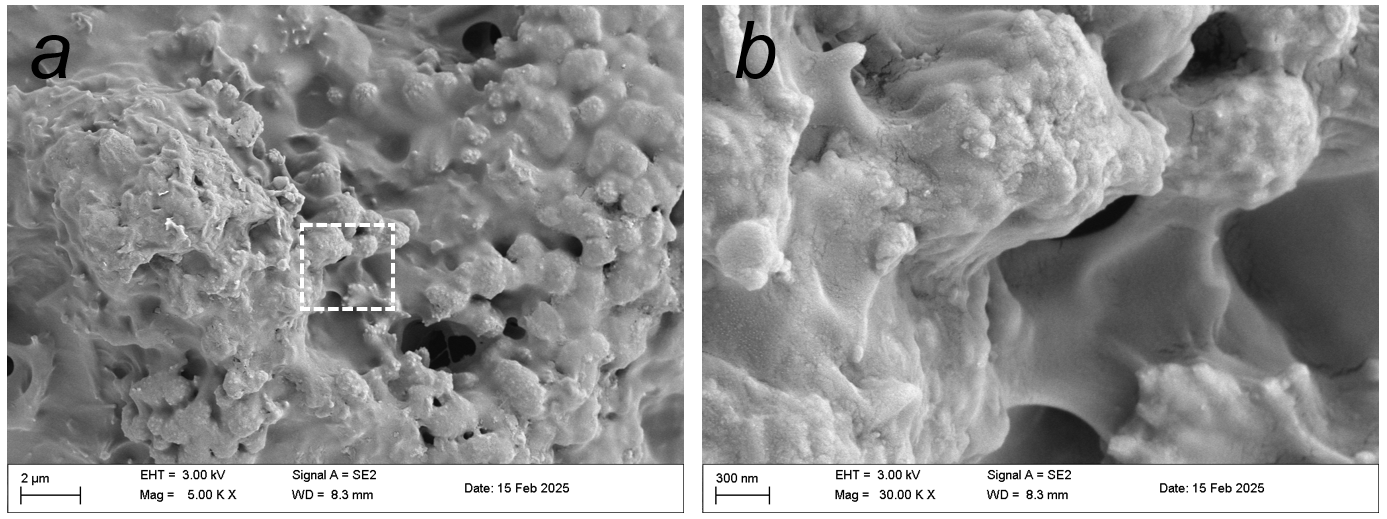


**Figure S7.** SEM images of P0.2HS1. (b) is the zoomed image of the box region of (a). CB clusters with a diameter of approximately 1 μm can be observed. Each cluster consists of numerous CB particles with size of smaller than 100 nm. Smooth-surface organic hydrogel covers the surface of electrode microstructure, including CB and hydrates.


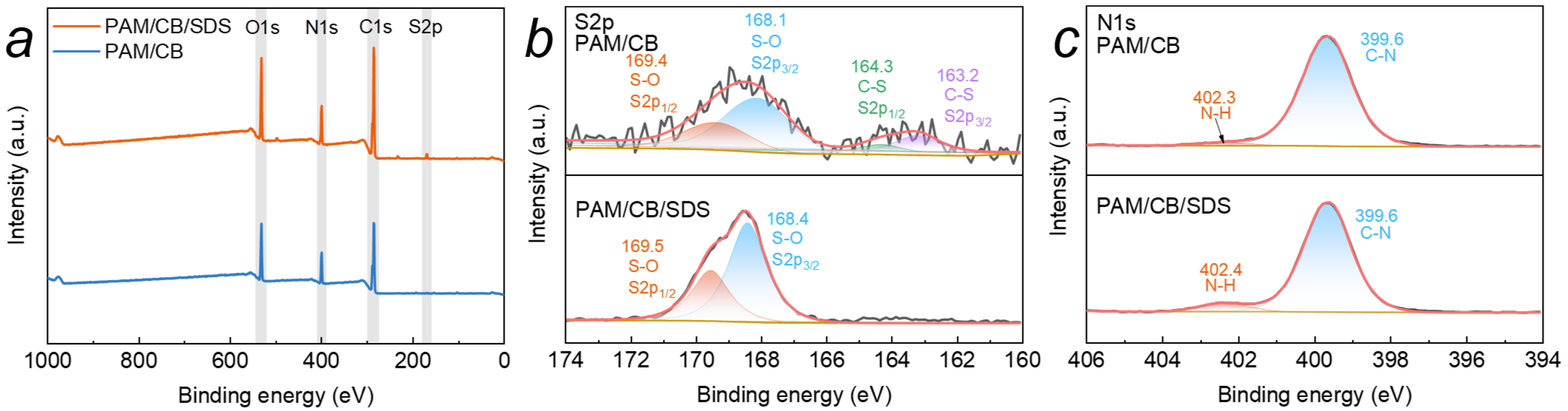


**Figure S8.** XPS spectra of CB before and after SDS modified in the PAM solutions. (a) The XPS survey spectra of CB/PAM suspension before and after SDS modification. Distinct peaks corresponding to S2p, C1s, N1s, and O1s are observed at binding energies of approximately 168 eV, 284 eV, 399 eV, and 532 eV, respectively [8-10]. (b) The high-resolution S2p spectra. In the reference sample, the S-O and C-S signals mainly originate from the initiator ammonium persulfate in the polyacrylamide solution. After SDS modification, noticeable changes are observed in both the intensity and binding energy positions of the S2p peaks, indicating the successful incorporation of sulfate groups. (c) The high-resolution N 1s spectra. A slight shift in the N-H peak position is detected after SDS treatment, suggesting a change in the chemical environment of the -NH_2_⁺ groups.


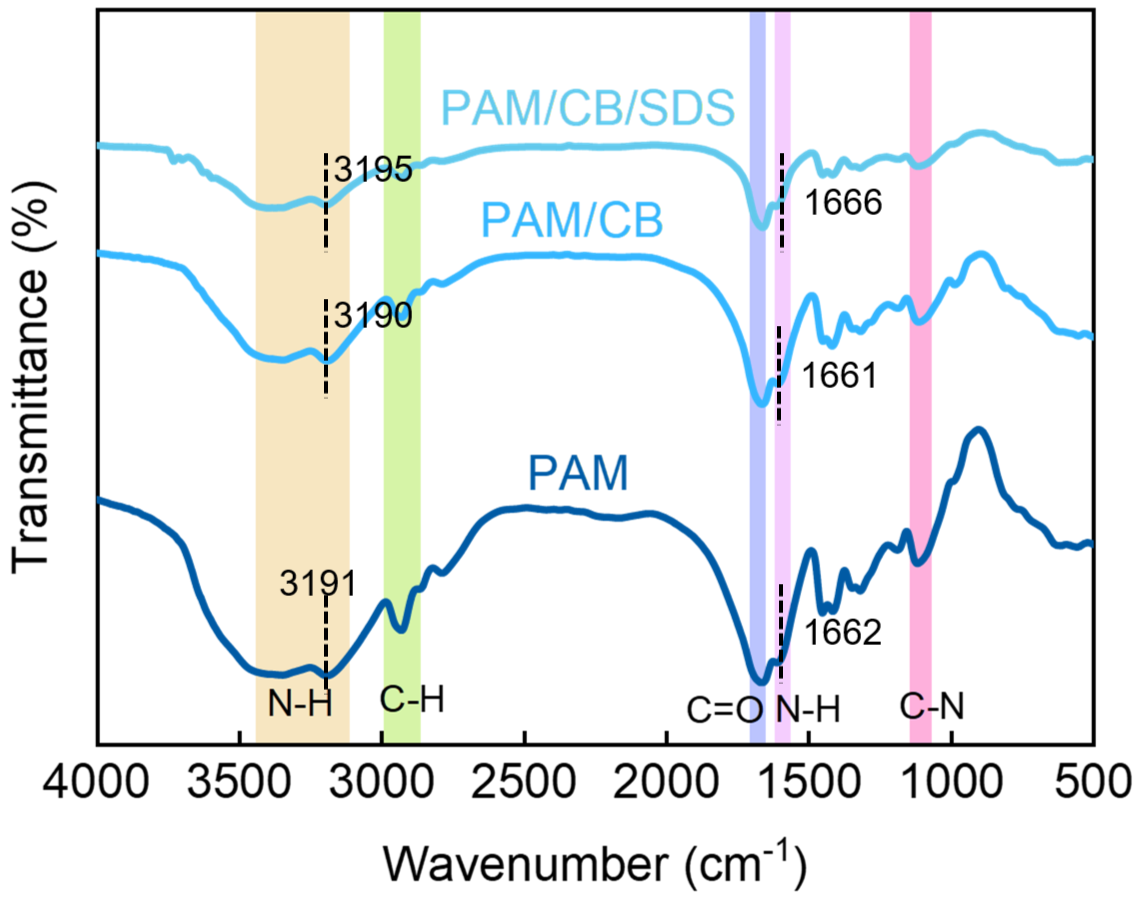


**Figure S9.** FTIR analysis results of CB before and after SDS modified. In the PAM suspensions, the peak at 2929 cm⁻¹ is attributed to the stretching vibration of C-H bonds. The first amide band corresponding to C=O stretching is observed at 1662 cm⁻¹, while the second one referring to C–N vibration appears at 1117 cm⁻¹ [11, 12]. Notably, in the PAM/CB/SDS spectrum, the bending vibration of the N-H bond shifts from 1661 cm⁻¹ to 1666 cm⁻¹. Additionally, the strong peak at 3191 cm⁻¹, assigned to the stretching vibration of N–H bonds and hydrogen bonding, shifts to 3195 cm⁻¹ [11, 12]. This shift is attributed to the formation of hydrogen bonds between the -NH_2_ of PAM and the -OSO_3_^-^ of SDS.


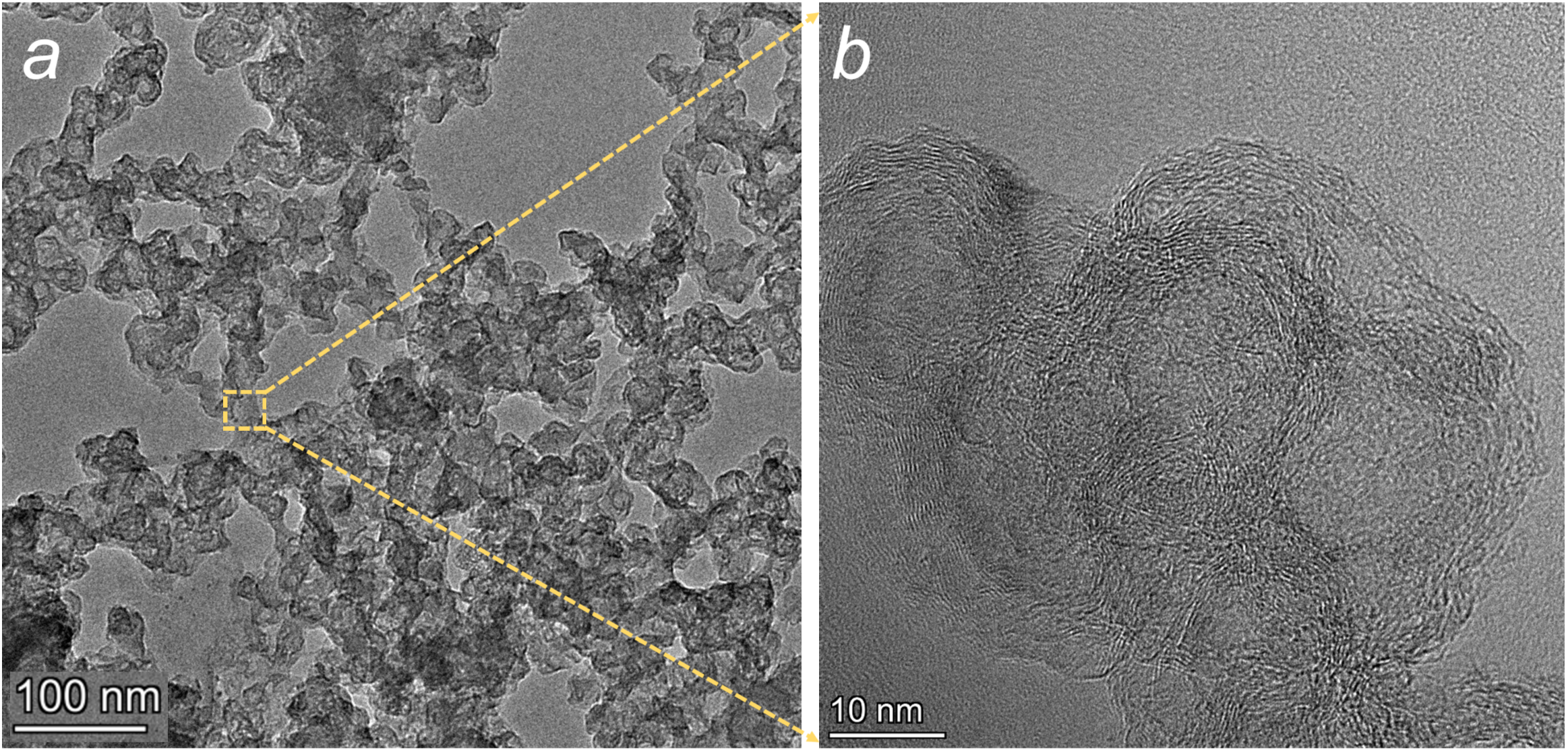


**Figure S10.** TEM image of CB/PAM suspension without SDS.


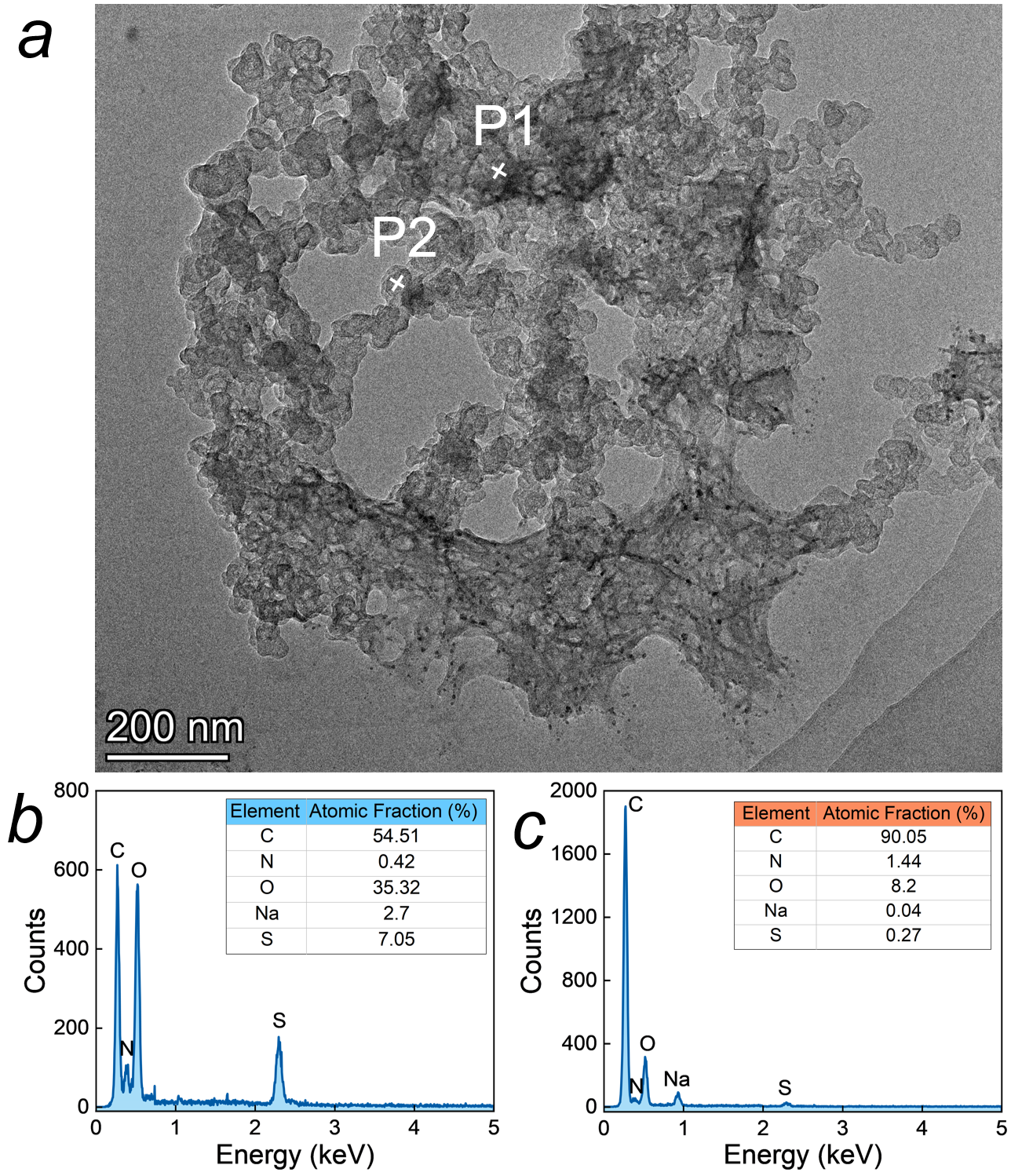


**Figure S11.** (a) TEM image and (b, c) EDS test of SDS-modified CB/PAM suspension solutions. The wrinkled PAM hydrogel encapsulates the particulate CB, generating the network configuration composed of the connected CB and PAM. The EDS results of (b) and (c) correspond the point P1 and P2, respectively, in the (a). The carbon content at point P2 exceeds 90%, which is the CB regions. At the point P1, the carbon content sharply drops to 54.51%, and there is 7.05% sulfur. It means the presence of SDS and PAM around CB particles.

**S3.3 Supplementary graph of CC electrode materials**


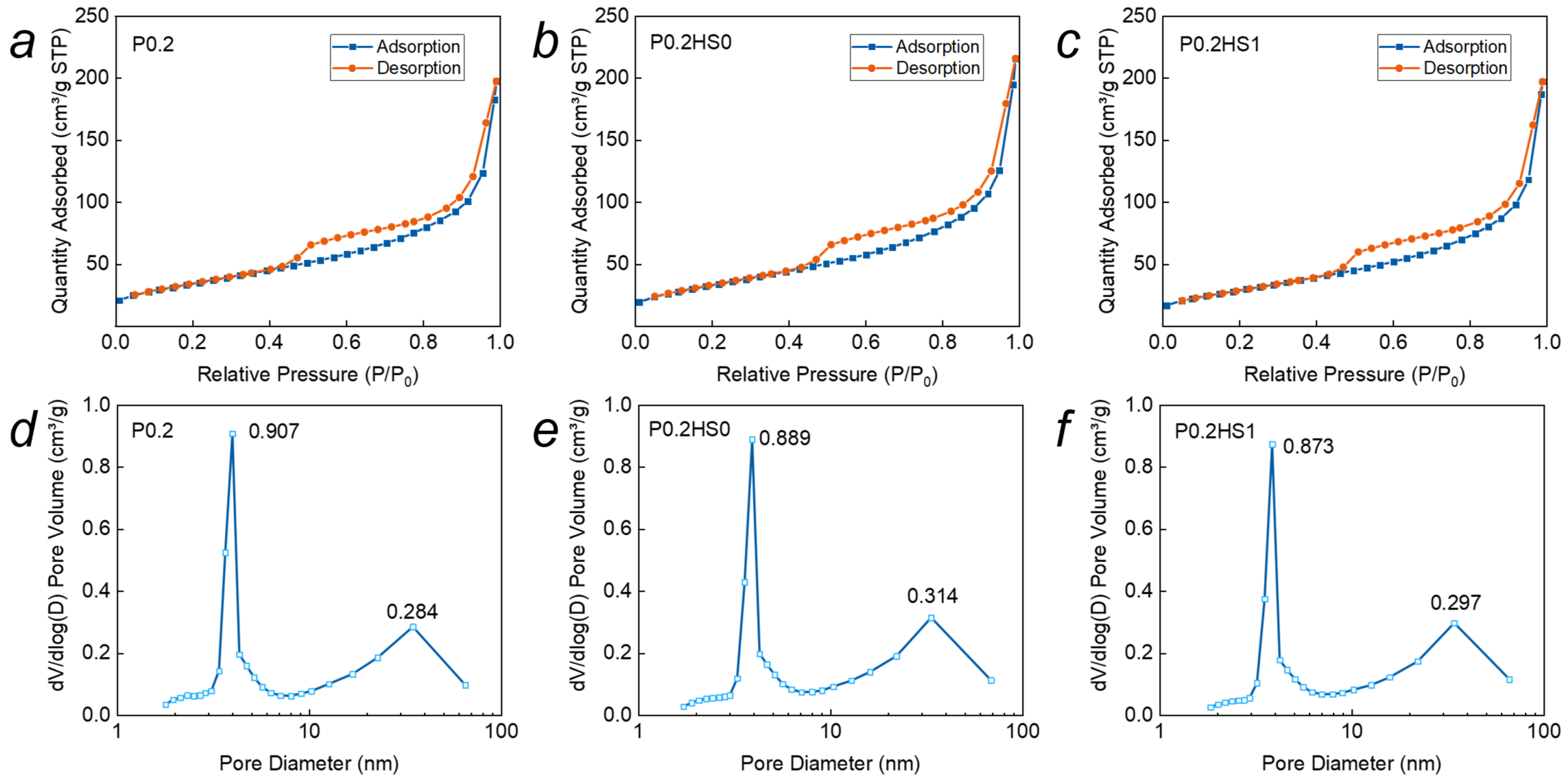


**Figure S12.** Adsorption desorption curves and pore diameter distributions of CC electrodes materials. (a)–(c) Adsorption–desorption curves obtained from BET. (d)–(f) Pore size distributions derived from BET. Sample P0.2, P0.2HS0, and P0.2HS1 exhibits hysteresis loops and most probable pore diameter similar to those of the pristine CB (Figure S4), indicating that the in-situ polymerization of PAM has almost no impact on the pore structure of electrode.

**Figure S13.** Volume of CC electrode sheets under different forming pressures. As compared with the casting formation, hot-stress formation significantly reduces the volume of the electrode. As the forming pressure increases, the volume of the P8 electrode sheet decreases to 1.12 cm³ (2.5 cm in diameter and 0.23 cm in thickness), indictive of the 30.4% reduction compared to P0.2 (2.5 cm in diameter and 0.3 cm in thickness).

**Figure S14.** CB interparticle distance of CC electrode sheets under different pressures. Assuming that CB particles are uniformly distributed spherical particles [13, 14], the interparticle distance (L) in the CC electrode was calculated using equation (), where d is the average diameter (derived from BET tests, d=8.68 nm) of CB particles and is the volume fraction of CB particles in the CC electrode. With increasing forming pressure, the average interparticle distance of CB decreases by 27.8%, from 9 nm to 6.5 nm.


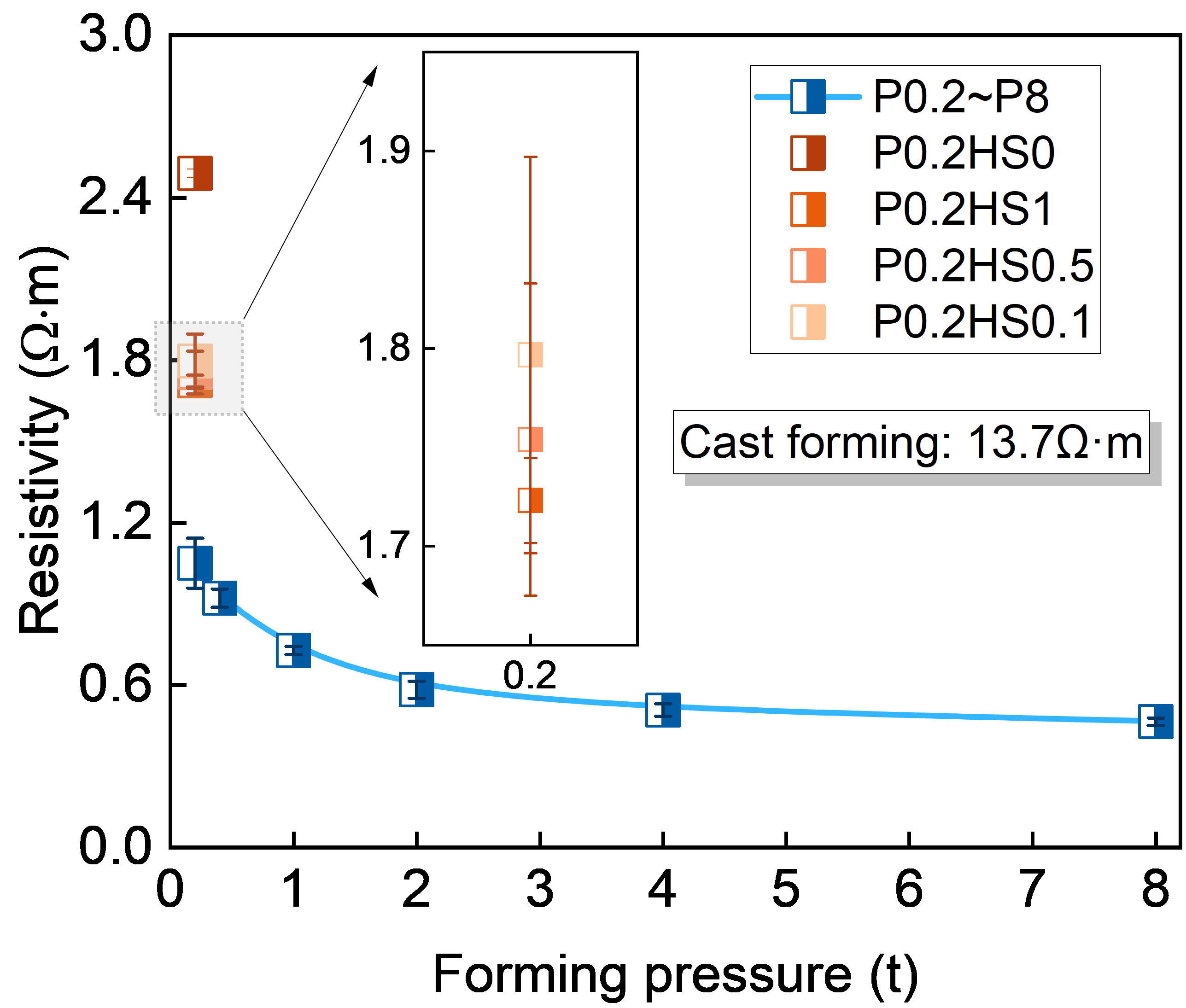


**Figure S15.** Resistivity of dried electrode materials under different pressures and SDS content. The resistivity drops with an increment in forming pressure, which implies the improved CB particles connectivity caused by the enhanced forming pressure. Electrode’s resistivity increases by 120% (1.15 Ω·m → 2.53 Ω·m) as a result of the MSHN formation in the electrode, and decreases as SDS content increases. It can be attributed to the improved dispersion of CB induced by SDS modification.


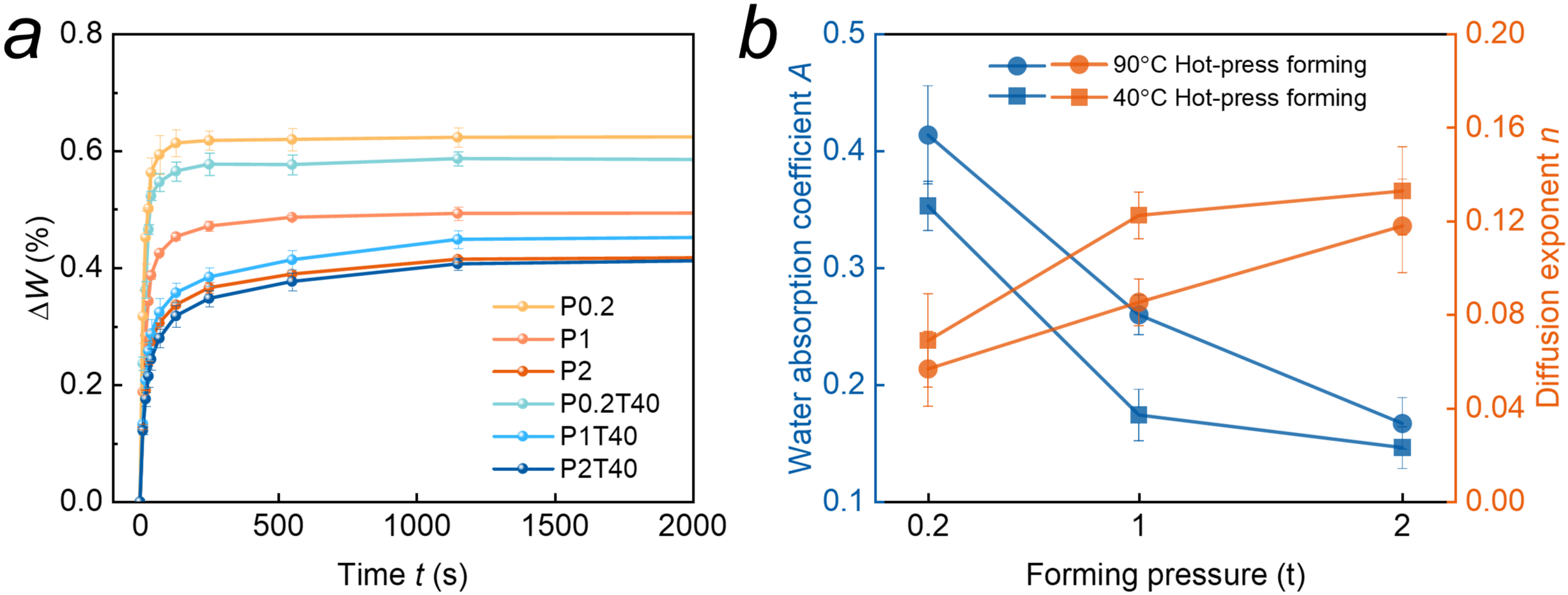


**Figure S16.** Water absorption performance of the CC electrode at different forming temperature and pressure. (a) The evolution of water absorption quantity $\Delta W$ of CC electrode with time. All specimens reach the plateau at approximately 1200 s. The 90 °C hot-press specimens exhibit the higher water absorption rate and quantity, indicating the higher porosity and pores connectivity. The water absorption coefficient (A) and diffusion coefficient (n) were obtained by fitting using the $\Delta W=A\cdot t^{n}$ equation [15-17]. The results are shown in (b). The water absorption coefficient of the sample formed by 90 °C hot-press is higher than that by 40°C.

**Figure S17.** Comparison of CC electrode materials prepared using different forming methods. In our previous study [18], the electrode material was cast with the water-cement ratio of 1.6, resulting the porosity of 54.5% and the compressive strength of 3.2 MPa. In contrast, the hot-press formation used in this study allowed for reduced water/cement ratio while maintaining the comparable porosity of 49.3%, and significantly improved the compressive strength to 8 MPa. This enhancement is attributed to the 90 °C hot-press process, which provides a highly efficient hydration environment and continuous steam pathways that facilitate pore channel development within the cement matrix.


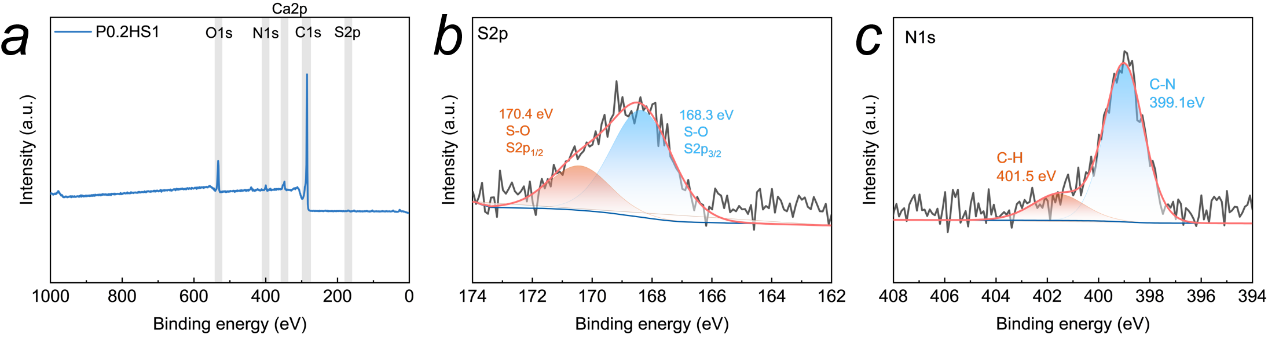


**Figure S18.** XPS spectra of P0.2HS1 electrode. (a) The XPS survey spectra. (b) The high-resolution S2p spectra. (c) The high-resolution N 1s spectra. XPS characterization was performed on the hardened electrodes. The survey spectrum clearly shows the presence of Ca 2p, N 1s, and S 2p peaks. Figure S18b presents the high-resolution S 2p spectrum, where the spin–orbit split peaks, S 2p₃/₂ and S 2p₁/₂, appear at 168.3 and 170.4 eV, respectively, characteristic of S-O bonds. The N 1s peak (Figure S18c) at 399.1 eV corresponds to the amino groups on the PAM chains.

**S3.4 Supplementary graph of electrochemical performance CC supercapacitors**


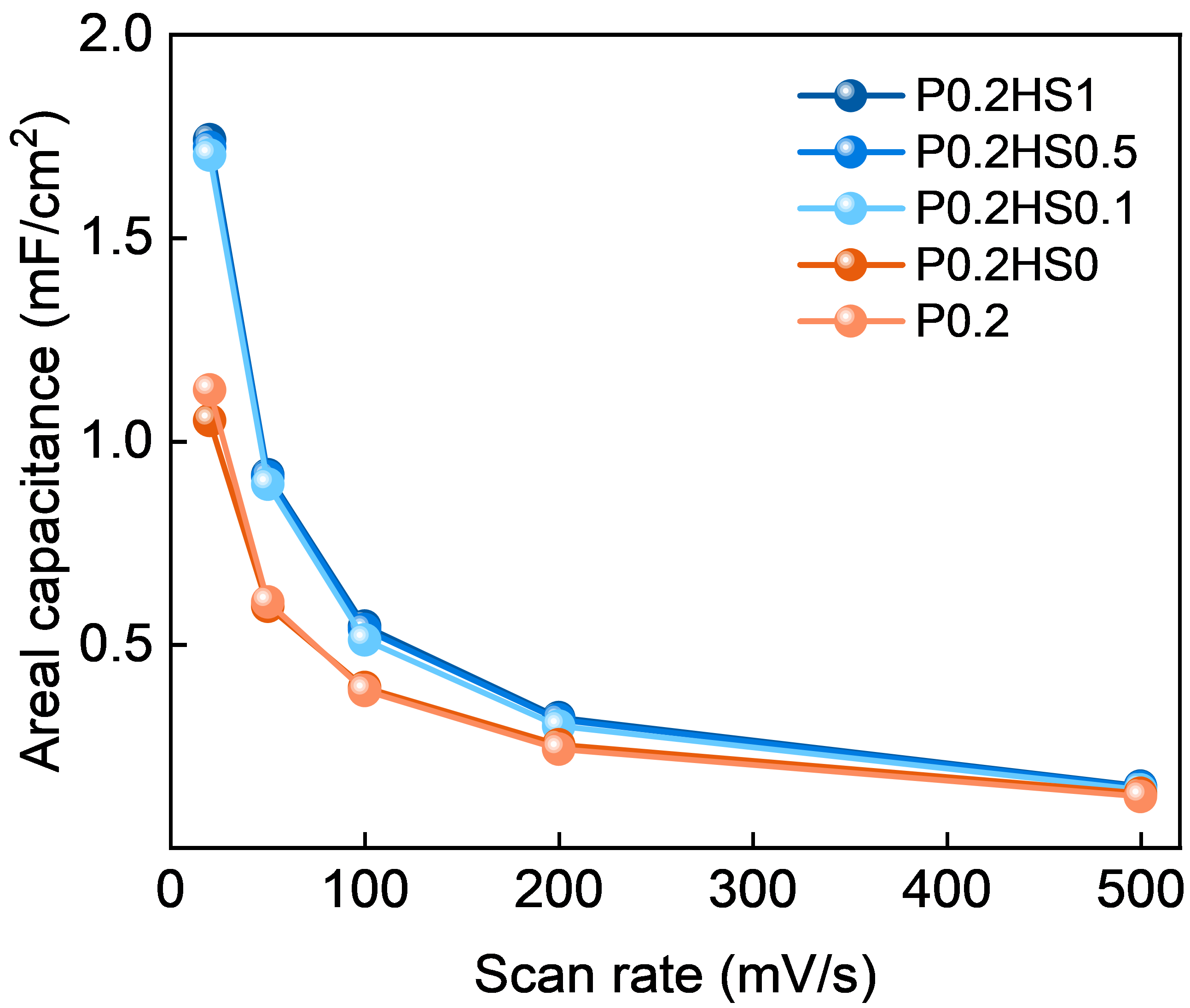


**Figure S19.** Rate performance of CV test with various scan rate.


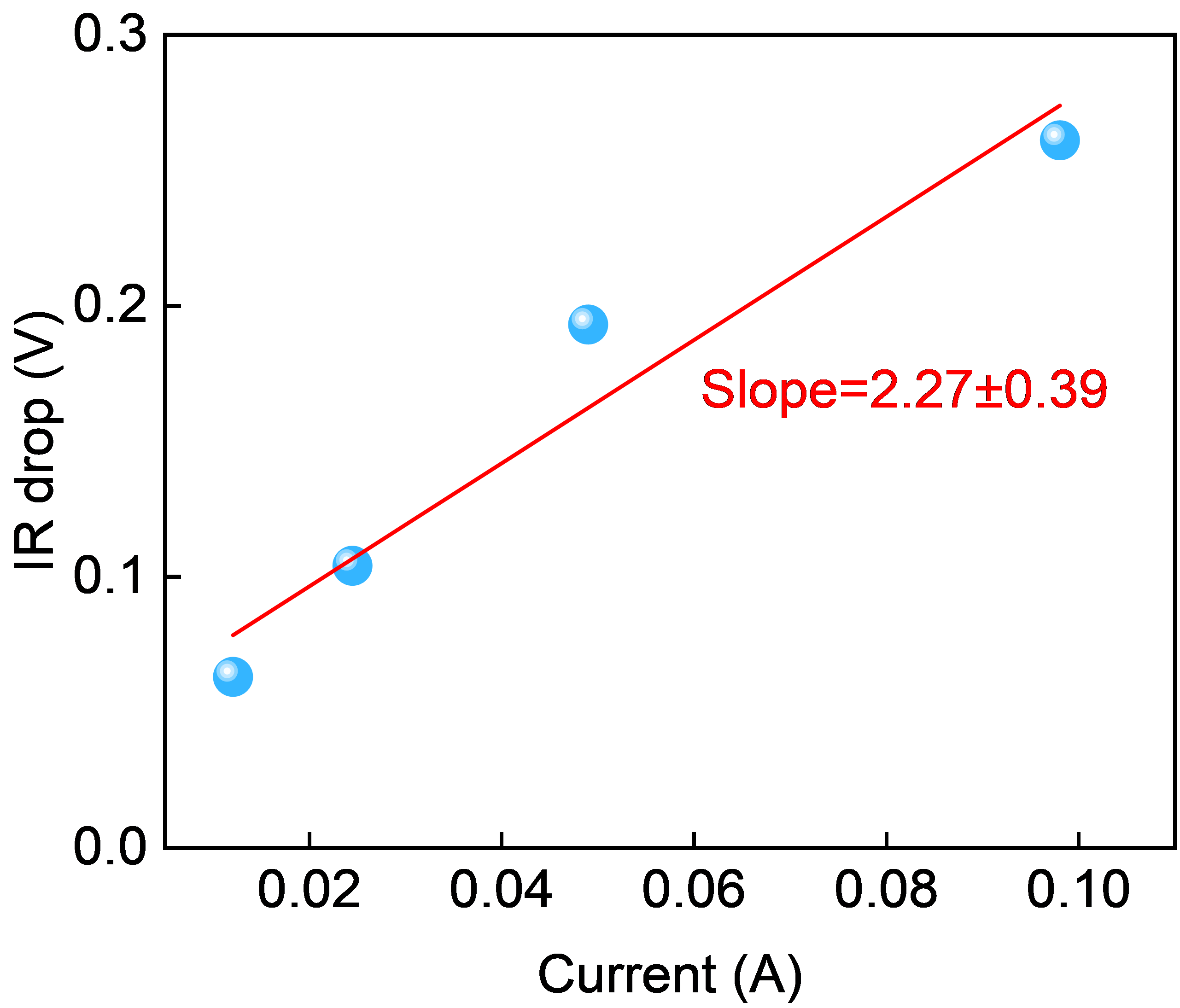


**Figure S20.** Relationship of IR drop and current of the GCD tests of P0.2HS1. The slope was measured to be 2.27 Ω, which is comparable to the results obtained from electrochemical impedance spectroscopy (EIS), ranging from 0.9 to 2.2 Ω.

**Table S5 EIS equivalent circuit fitting**

|  | ESR  (Ω) | CPE-T | CPE-P | R_C_  (Ω) | W-R  (Ω·s^0.5^) | W-T  (s) | W-P | D  (m^2^/s) |
| --- | --- | --- | --- | --- | --- | --- | --- | --- |
| P0.2HS1 | 0.898 | 0.029 | 0.679 | 0.112 | 2.370 | 21.700 | 0.478 | 7.373×10^-7^ |
| P0.2HS0.5 | 0.916 | 0.020 | 0.733 | 0.094 | 3.206 | 23.510 | 0.478 | 6.806×10^-7^ |
| P0.2HS0.1 | 0.936 | 0.023 | 0.641 | 0.197 | 3.351 | 15.410 | 0.437 | 1.038×10^-6^ |
| P0.2HS0 | 1.369 | 0.420 | 0.331 | 0.281 | 7.890 | 57.800 | 0.458 | 2.768×10^-7^ |
| P0.2 | 0.950 | 0.031 | 0.645 | 0.119 | 8.566 | 71.590 | 0.477 | 2.235×10^-7^ |
| P0.4 | 1.357 | 0.045 | 0.493 | 0.139 | 7.352 | 70.430 | 0.505 | 1.719×10^-7^ |
| P1 | 1.811 | 0.155 | 0.657 | 0.164 | 23.990 | 149.100 | 0.527 | 6.445×10^-8^ |
| P2 | 1.690 | 0.117 | 0.584 | 0.131 | 20.110 | 172.220 | 0.494 | 4.883×10^-8^ |
| P4 | 1.859 | 0.153 | 0.748 | 0.095 | 21.750 | 181.660 | 0.477 | 4.316×10^-8^ |
| P8 | 2.257 | 0.011 | 0.831 | 0.081 | 27.890 | 217.800 | 0.458 | 3.104×10^-8^ |


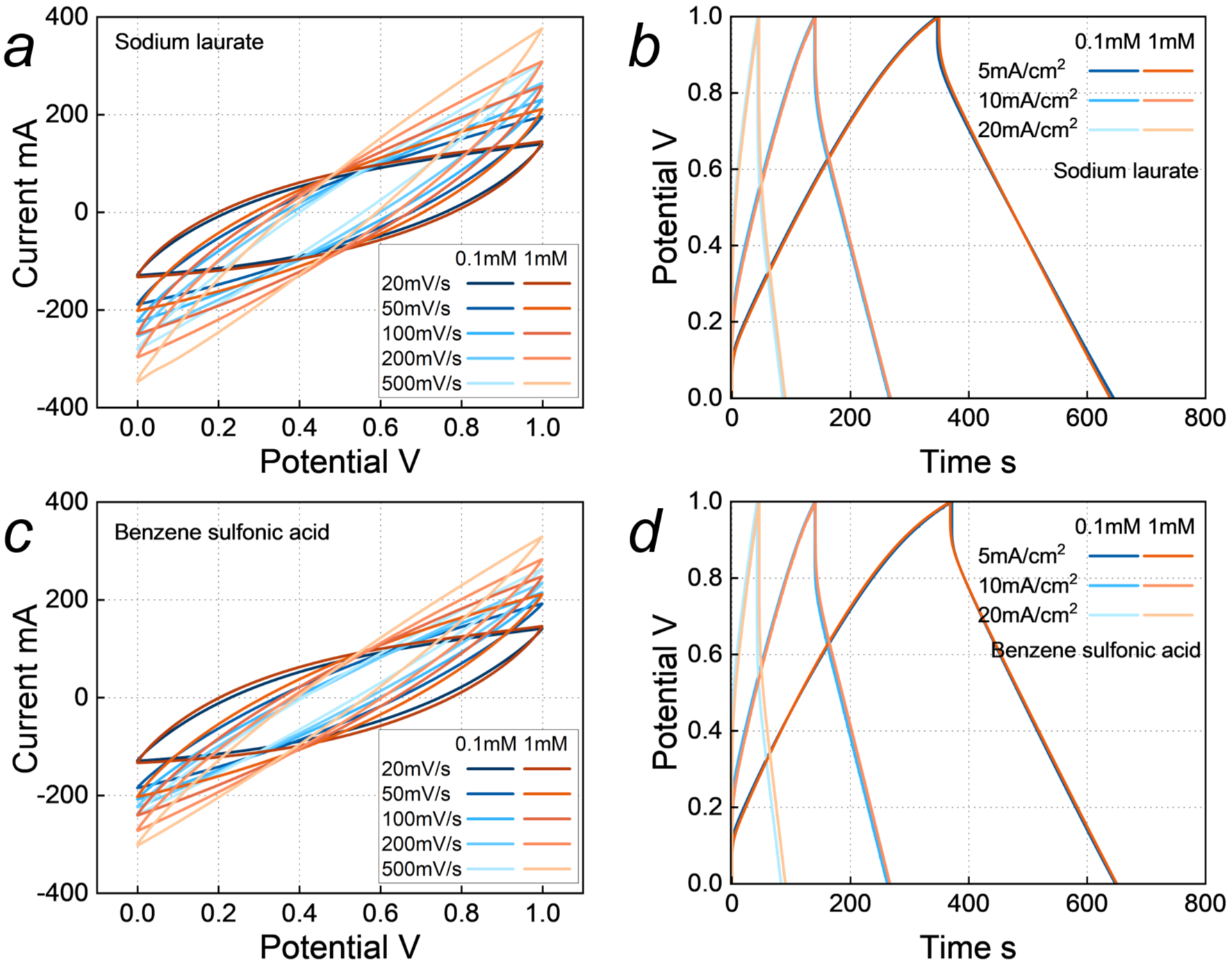


**Figure S21.** CV and GCD curves of the specimens with sodium laurate or benzenesulfonic acid.

**Figure S22.** CV curves of P0.2, P0.4, P1, P2, P4, and P8.

**Figure S23.** GCD curves of P0.2, P0.4, P1, P2, P4, and P8.

**Figure S24.** Power density and energy density of CCS.

**Figure S25.** Comparsions of areal capacitance from relative reference.

**Table S6 Compressive strength and areal capacitance of this work versus similar reports**

| Electrode | Electrolyte | Compressive strength (MPa) | Specific Cap. (mF/cm^2^) | Energy density | Cycle performance | Ref. |
| --- | --- | --- | --- | --- | --- | --- |
| Carbon-cement electrode with 12.5% KB | 1M KCl | 4-31.2 | 1708 901 | 395Wh/m^3^  (0.316mWh/cm^2^)  (3.61Wh/kg) | 10^4^＞83% | This work |
| Carbon-cement electrode with 15% KB | 1M KCl | 25 | 761.7 | 0.08mWh/cm^2^ | -- | 19 |
| Carbon-cement electrode with 12.8% KB | 1M KCl |  | 940* | 220Wh/m^3^  20Wh/m^3^ |  | 20 |
| CuCo2O4/rGO as positive electrode rGO@Ni foam as negative electrode | Polyethylene oxide cement-5%KOH | 7.8 | 439.35 | 0.2mWh/cm^2^ | 10^4^＞85% | 21 |
| CuCo2O4/rGO as positive electrode rGO@Ni foam as negative electrode | SPolyethylene oxide cement 5%LiOTf | 9.2 | 407.07 | 0.74mWh/cm2 |  | 21 |
| rGO@MnO2 as the positive electrode  rGO as the negative electrode | Polyacrylic acid Portland cement-2M KOH | 28.5 | 51.5 |  |  | 22 |
| rGO/Ni foam electrode | Polyacrylamide/alkali-activated slag-2M KOH | 44.5(dry)  36.9(soaked) | 254.4 | 2.48Wh/kg | 5000>86% | 23 |
| rGO/Ni foam electrode | Portland cement SSP-GP | 33.8–47.2 | 18.7–22 |  |  | 24 |
| rGO/Ni foam electrode | Cement 2wt%-Na2SO | 32.67 | 35.18 |  | 5000>92% | 25 |
| rGO/Ni foam electrode | Foamed porous cement-2M KOH | 19.6 | 178.28 | 14Wh/kg | 2000>90% | 26 |
| rGO/CuO | PAA-PC-LiOTf electrolyte | 23.65 | 314.9 | 0.41mWh/cm2 | 5000>97% | 27 |
| rGO/Fe2O3 electrode | Redox active cement-2M KOH | 12~13 | 166 | 0.052mWh/cm2 | 2000>80% | 28 |
| Carbon fiber | Geopolymer cement electrolyte | 43 | 86 | 0.0172mWh/cm2 |  | 29 |
| Ni foam CB | 3D print cement-2M KOH | 32.5 | 1.59 |  |  | 30 |
| rGO/Ni foam electrode | H_2_O_2_ foaming cement-2M KOH | 18.1 | 351.5 | 21.6Wh/kg | 5000>91% | 31 |
| rGO/Ni foam electrode | PAA cement-KOH | 16.2 | 31.88 | 15.93mWh/cm2 | 10000>84% | 32 |

^*^ Although the specific areal capacitance is not directly reported in the article [20], it can be calculated from the given total capacitance (3.57 F) and the electrode diameter (2.2 cm). It is approximately 940 mF/cm².

**Figure S26.** Performance recovery of P0.2HS1 after re-soaking and it can still works for 20,000 charge-discharge cycles.


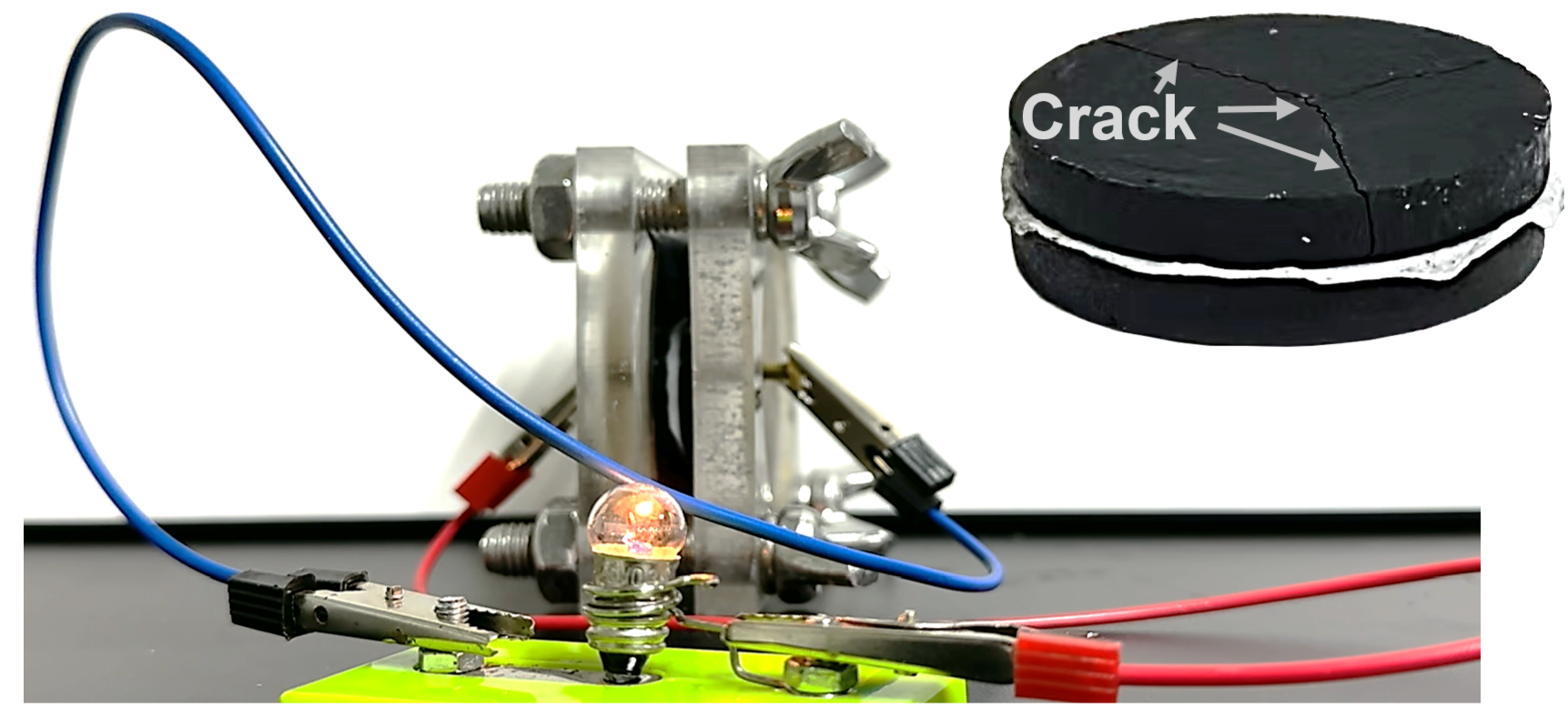


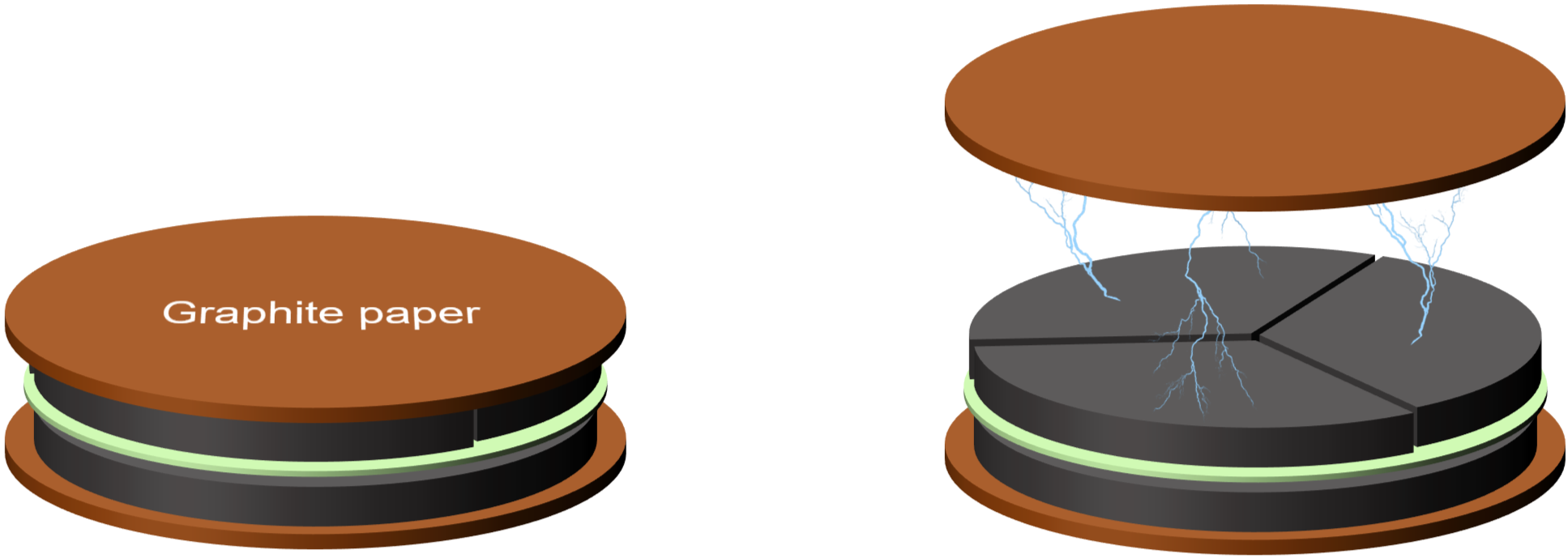


**Figure S27.** CC supercapacitor with three through cracks lights a bulb.


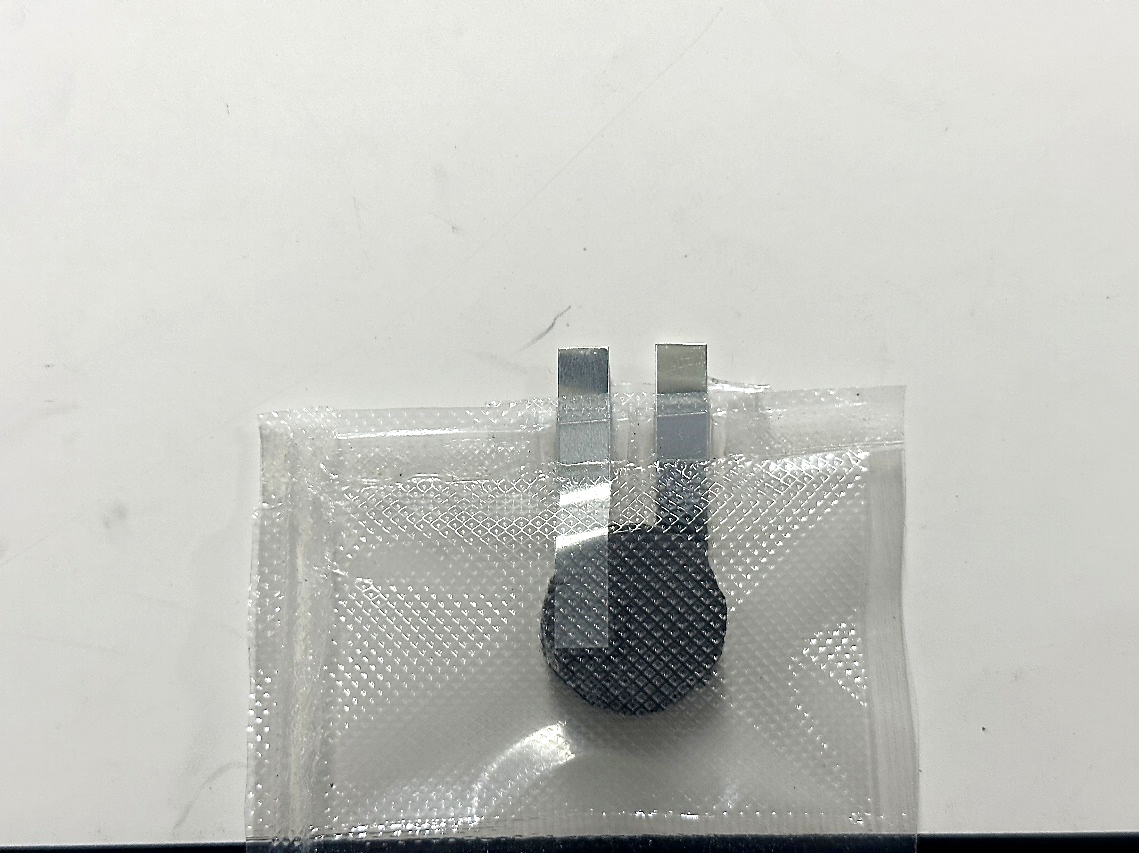


**Figure S28.** Simple package of the CCS for the test at different temperature.


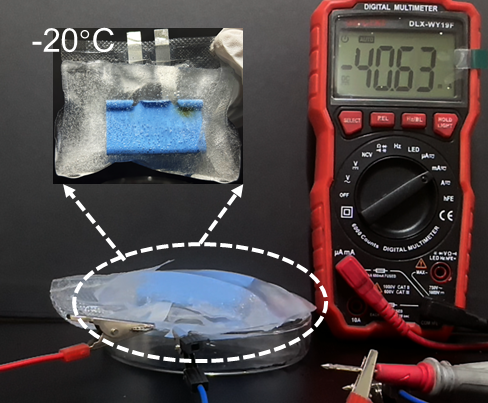


**Figure S29.** Frozen test of CC supercapacitor.


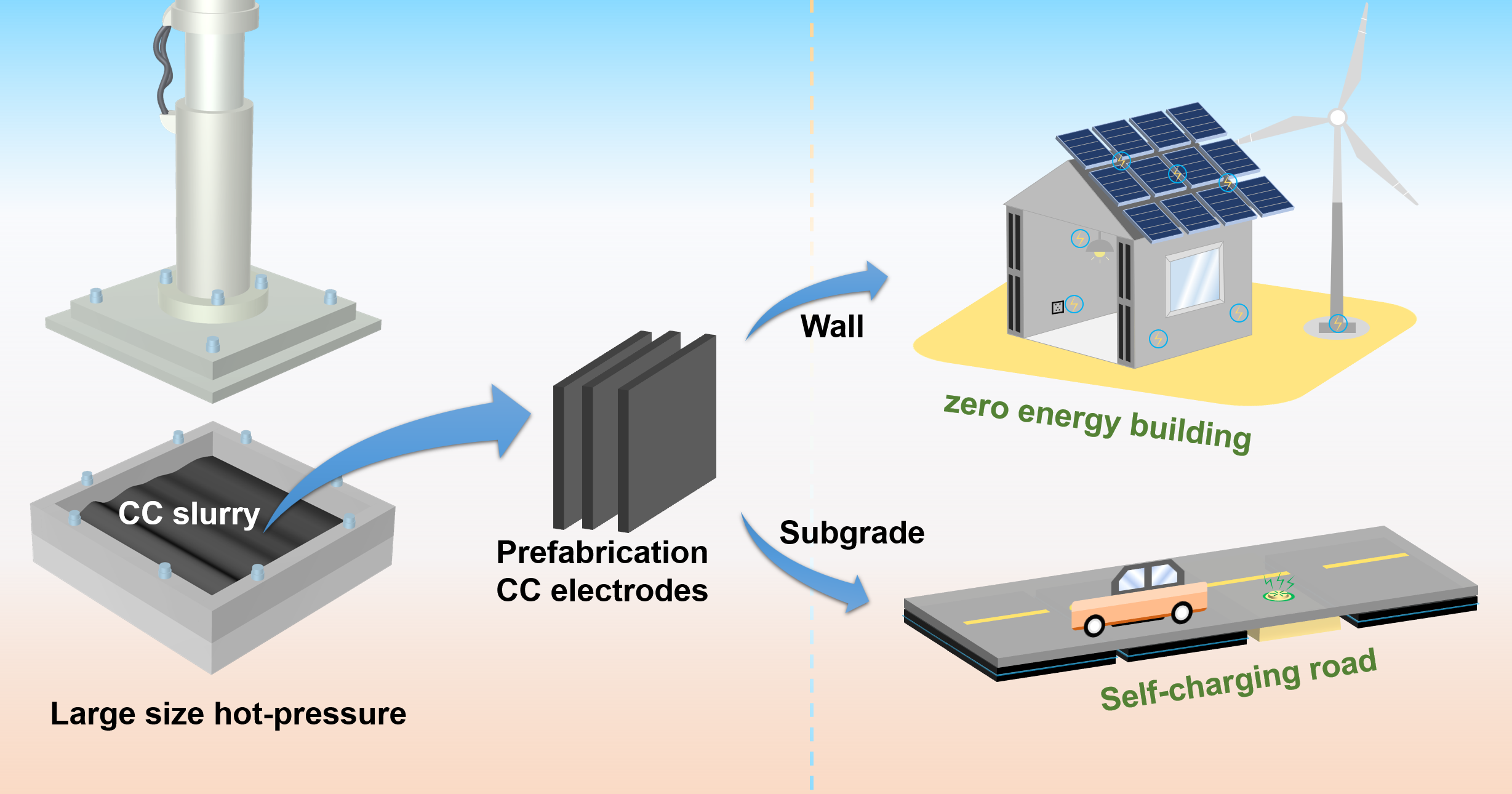


**Figure S30.** Preparation and future application of large CC electrodes.

**Reference**

1. Hakimi M, Hakimi M. Effect of activated Ketjen black and nano-size sulfur particles on electrochemical performance of lithium-sulfur battery. Colloids and Surfaces A: Physicochemical and Engineering Aspects. 2024;685:133265.
2. Kang J, Li O L, Saito N. Hierarchical meso–macro structure porous carbon black as electrode materials in Li–air battery. Journal of Power Sources. 2014;261:156-61.
3. Amin A S, Caidi A, Lange T, et al. Key Control Characteristics of Carbon Black Materials for Fuel Cells and Batteries for a Standardized Characterization of Surface Properties. Particle & Particle Systems Characterization. 2025;42(1):2400069.
4. Knight D S, White W B. Characterization of diamond films by Raman spectroscopy. Journal of Materials Research. 1989;4(2):385-93.
5. Ye Y, Wu F, Liu Y, et al. Toward Practical High-Energy Batteries: A Modular-Assembled Oval-Like Carbon Microstructure for Thick Sulfur Electrodes. Advanced Materials. 2017;29(48):1700598.
6. Picquart M. Vibrational model behavior of SDS aqueous solutions studied by Raman scattering. The Journal of Physical Chemistry. 1986;90(2):243-50.
7. Karlsson P M, Palmqvist A E C, Holmberg K. Adsorption of Sodium Dodecyl Sulfate and Sodium Dodecyl Phosphate on Aluminum, Studied by QCM-D, XPS, and AAS. Langmuir. 2008;24(23):13414-19.
8. Lima M C F S, Zaida do Amparo S, Ribeiro H, et al. Aqueous suspensions of carbon black with ethylenediamine and polyacrylamide-modified surfaces: Applications for chemically enhanced oil recovery. Carbon. 2016;109:290-99.
9. Chen X Y, Chen C, Zhang Z J, et al. Nitrogen-Doped Porous Carbon Spheres Derived from Polyacrylamide. Industrial & Engineering Chemistry Research. 2013;52(34):12025-31.
10. Viltres H, Odio O F, Biesinger M C, et al. Preparation of Amine- and Disulfide-Containing PAMAM-Based Dendrons for the Functionalization of Hydroxylated Surfaces: XPS as Structural Sensor. ChemistrySelect. 2020;5(16):4875-84.
11. Dragan E S, Perju M M, Dinu M V. Preparation and characterization of IPN composite hydrogels based on polyacrylamide and chitosan and their interaction with ionic dyes. Carbohydrate Polymers. 2012;88(1):270-81.
12. Chen Y, Zhao Y, Zhou S, et al. Preparation and characterization of polyacrylamide/palygorskite. Applied Clay Science. 2009;46(2):148-52.
13. Lux F. Models proposed to explain the electrical conductivity of mixtures made of conductive and insulating materials. Journal of Materials Science. 1993;28(2):285-301.
14. Jean-Baptiste D. Carbon black: science and technology. CRC Press, 1993.
15. Wang P, Dong S, Zhang Y, et al. Investigation of the Penetration of Water and Chloride into Unsaturated Concrete: An Experimental and Molecular Dynamics Study. Journal of Materials in Civil Engineering. 2024;36(12):04024409.
16. Rucker-Gramm P, Beddoe R E. Effect of moisture content of concrete on water uptake. Cement and Concrete Research. 2010;40(1):102-08.
17. Kameche Z, Ghomari F, Choinska M, et al. Assessment of liquid water and gas permeabilities of partially saturated ordinary concrete. Construction and Building Materials. 2014;65:551-65.
18. Li P, Liu Q, Jiang J, et al. Capacitance and coulombic efficiency controlling of cement-based supercapacitors. Journal of Energy Storage. 2025;130:117446.
19. Yan D, Mao J, Gao R, et al. Improving the electrochemical performance of cement-based supercapacitors through microstructure optimization. Journal of Energy Storage. 2024;96:112717.
20. Chanut N, Stefaniuk D, Weaver J C, et al. Carbon–cement supercapacitors as a scalable bulk energy storage solution. Proceedings of the National Academy of Sciences. 2023;120(32):e2304318120.
21. Fang C, Zhang D. High multifunctional performance structural supercapacitor with Polyethylene oxide cement electrolyte and reduced graphene oxide@CuCo2O4 nanowires. Electrochimica Acta. 2022;401:139491.
22. Fang C, Zhang D. A large areal capacitance structural supercapacitor with a 3D rGO@MnO2 foam electrode and polyacrylic acid–Portland cement–KOH electrolyte. Journal of Materials Chemistry A. 2020;8(25):12586-93.
23. Ran M, Wang J, Zhang D. A superior electrolyte composite with high conductivity and strength based on alkali-activated slag and polyacrylamide for structural supercapacitor. Journal of Energy Storage. 2024;79:110169.
24. Zhan P, Xu J, Wang J, et al. Structural supercapacitor electrolytes based on cementitious composites containing recycled steel slag and waste glass powders. Cement and Concrete Composites. 2023;137:104924.
25. Fang C, Zhang D. Portland cement electrolyte for structural supercapacitor in building application. Construction and Building Materials. 2021;285:122897.
26. Zhou C, Wang Q, Zhang C. Electrochemical Energy Storage Properties of High-Porosity Foamed Cement. Materials. 2022;15(7):2459.
27. Fang C, Zhang D. High areal energy density structural supercapacitor assembled with polymer cement electrolyte. Chemical Engineering Journal. 2021;426:130793.
28. Wang J, Zhan P, Zhang D. Redox active cement-based electrolyte towards high-voltage asymmetric solid supercapacitor. Cement and Concrete Composites. 2023;138:104987.
29. Zhu J-H, Wang X, Yu H, et al. Advanced industrial-grade carbon-fiber-reinforced geopolymer cement supercapacitors for building-integrated energy storage solutions. Cement and Concrete Composites. 2025;161:106106.
30. Lyu Q, Wang Y, Chen D, et al. Energy storage properties and mechanical strengths of 3D printed porous concrete structural supercapacitors reinforced by electrodes made of carbon-black-coated Ni foam. Cement and Concrete Composites. 2025;157:105926.
31. Shi M, Zhang D. Integrated construction improving electrochemical performance of loadable supercapacitors based on porous cement-based solid electrolytes. Journal of Power Sources. 2024;616:235135.
32. Wang J, Xu C, Zhang D, et al. Porous polymer cement composites for quasi-solid graphene supercapacitors. Journal of Energy Storage. 2023;63:106991.
